# Development of internalizing symptoms during adolescence in three countries: the role of temperament and parenting behaviors

**DOI:** 10.1007/s00787-021-01725-6

**Published:** 2021-02-06

**Authors:** Carolina Lunetti, Anne-Marie R. Iselin, Laura Di Giunta, Jennifer E. Lansford, Nancy Eisenberg, Concetta Pastorelli, Dario Bacchini, Liliana Maria Uribe Tirado, Eriona Thartori, Emanuele Basili, Irene Fiasconaro, Ainzara Favini, Maria Gerbino, Flavia Cirimele, Chiara Remondi, Ann T. Skinner, W. Andrew Rothenberg

**Affiliations:** 1 Department of Psychology, Sapienza University of Rome, Rome, Italy; 2 Psychology Department, Elon University, Elon, USA; 3 Center for Child and Family Policy, Duke University, Durham, USA; 4 Psychology Department, Arizona State University, Tempe, USA; 5 Psychology Department, Universidad San Buenaventura, Bogota, Colombia; 6 Psychology Department, II Second University of Naples, Naples, Federico Italy

**Keywords:** Emotionality, Effortful control, Temperament, Parenting, Internalizing, Adolescence

## Abstract

This longitudinal study examined the unique and joint effects of early adolescent temperament and parenting in predicting the development of adolescent internalizing symptoms in a cross-cultural sample. Participants were 544 early adolescents (T1: *M*_age_ = 12.58; 49.5% female) and their mothers (*n* = 530) from Medellín, Colombia (*n* = 88), Naples, Italy (*n* = 90), Rome, Italy (*n* = 100) and Durham, North Carolina, United States (African Americans *n* = 92, European Americans *n* = 97, and Latinx *n* = 77). Early adolescent negative emotionality (i.e., anger and sadness experience), self-regulation (i.e., effortful control), and parent monitoring and psychological control were measured at T1. Adolescent internalizing symptoms were measured at three time points. Latent Growth Curve Modeling (LGCM) without covariates or predictors indicated a slight linear increase in internalizing symptoms from ages 13–16 years across nearly all cultural groups. Multi-group LGCMs demonstrated several paths were consistently invariant across groups when examining how well temperament and parenting predicted intercept and slope factors. Higher initial levels of internalizing symptoms were significantly predicted by higher adolescent negative emotionality and parental psychological control as well as lower adolescent effortful control and parental monitoring measured one year earlier. Overall, adolescent effortful control appeared to protect against the emergence of internalizing symptoms in all cultures, but this effect faded over time. This study advances knowledge of the normative development of internalizing symptoms during adolescence across cultures while highlighting the predictive value of early adolescent temperament and parenting.

**Supplementary Information:**

The online version contains supplementary material available at 10.1007/s00787-021-01725-6.

## Introduction

Transitioning to adolescence is associated with challenges related to changes in biological, cognitive, emotional, and social systems [48]. How adolescents face those challenges influences their psychological adjustment and long-term outcomes. Epidemiological research indicates mental health problems in adolescence are a principal cause of adolescent deaths worldwide [53]. To enhance the well-being of youth worldwide, it is important to investigate the development of mental health symptoms as well as parent- and child-based predictors of that development among adolescents from different cultures. Accordingly, the overall aim of this longitudinal study was to examine the unique and joint relations of early adolescent temperament and parenting to the development of adolescents’ internalizing symptoms in a cross-cultural sample. In this regard, empirical evidence shows that early adolescent temperament and parenting behaviors predict the development of internalizing symptoms during adolescence. However, cross-national longitudinal evidence on these relations is limited. Therefore, the present study is exploratory in nature, seeking to partially fill this gap in the literature.

## Development of internalizing symptoms

Epidemiological studies of internalizing symptoms suggest they are relatively stable during childhood and increase during adolescence (e.g., [52]). Galambos et al. [20] found that internalizing symptoms increased over a three-year period in early adolescence. Bongers et al. [10] found that internalizing problems grew linearly and quadratically from age 4 to 18 years, with steeper increases at younger ages. Among children aged 5–17 years, Leve et al. [26] found that girls’ internalizing symptoms increased over time, whereas boys’ internalizing symptoms remained stable. Among adolescents followed for five years from ages 13 to 18 years, Maciejewski et al. [31] found increases in overall negative mood and, specifically, in anxiety and sadness. Prior work examining cultures included in this study found that youth experienced slight decreases in internalizing symptoms from ages 8 to 10 (in all cultures except US Latinx^1^) or 8–12 (in Latinx), but experienced increases in internalizing symptoms thereafter until age 14. US African American youth’s internalizing symptoms decreased between ages 8 and 10 and then remained stable until age 14 [45]. With few exceptions, internalizing symptoms generally increase during adolescence across cultures (e.g., [4]). Given these findings, we hypothesized that internalizing symptoms would increase linearly from age 13 to 17 across countries, except for US African American adolescents whose internalizing symptoms may stabilize.

## Predicting the development of internalizing symptoms

### Child temperament

Temperament—defined as “the constitutionally based individual differences in reactivity and self-regulation, as seen in the emotional, motor, and attentional domains” [44], p. 357—predicts long-term adjustment (e.g., [18]). Temperament characteristics such as negative emotionality [i.e., frequency of negative emotions such as anger, fear, and sadness and effortful control (i.e., regulating attention, activating and inhibiting behavior can make children more vulnerable to mental health issues [36])]. Eisenberg et al. [18] found that school-aged internalizers were characterized by high negative emotionality. Among Dutch adolescents, Oldehinkel et al. [37] found that internalizing problems were more strongly associated with negative emotionality as compared to effortful control. Internalizing problems often involve dysregulation of negative emotions and behavioral inhibition, which are linked to poor effortful control [33]. Low effortful control could be related to the development of internalizing problems through the inability to direct attention away from negative emotions, thoughts, and rumination [56]. Furthermore, relations between effortful control and internalizing symptoms may not be direct. Among Dutch preadolescents, Oldehinkel et al. [38] found an attenuated effect of negative emotionality on internalizing problems in children with high effortful control.

In sum, previous studies mainly examining adolescents from the US or, more rarely, North European countries, suggest that high negative emotionality and its interaction with effortful control may be associated with internalizing problems. Cross-national longitudinal evidence is limited. However, extrapolating from related research on temperament and adjustment among children outside the US (e.g., [34]), we hypothesized that negative emotionality and effortful control (and their interaction) would be related to internalizing symptoms similarly across cultures.

### Parenting

Cross-sectional and some limited longitudinal evidence indicates that psychological control and parental monitoring (and perhaps their interaction) are related to internalizing symptoms. Psychological control refers to parental attempts to pressure a child through internally controlling means, including manipulation and intrusion into the child’s life through behaviors such as invalidating feelings and pressuring the child to think in particular ways using disappointment, guilt, and shame induction [7]. Cross-sectionally, psychological control is associated with higher internalizing symptoms [41], a finding that appears to hold across nations, such as Cypress [50], and Spain [40]. Longitudinal investigations suggest mixed findings. Lansford et al. [24] found that higher psychological control predicted increases in internalizing symptoms, whereas Galambos et al. [20] reported it did not.

Parental monitoring is defined as ‘‘parents’ knowledge of the child’s whereabouts, activities, and associations’’ [47], p. 1074. Cross-sectional evidence suggests parental monitoring is negatively associated with adolescents’ internalizing symptoms across cultural contexts (e.g., [5, 28, 51]). Longitudinal research on Dutch adolescents found that high parental monitoring predicted fewer internalizing symptoms two years later [54], whereas other longitudinal findings among American adolescents were either non-significant [12] or applied only to boys [24]. These mixed findings might be better understood by examining the interaction between psychological control and parental monitoring. For example, a cross-sectional study found that adolescents reporting the highest levels of internalizing symptoms had parents who were high in psychological control and low in parental monitoring [42].

While there are few cross-national longitudinal studies of relations between parenting and internalizing symptoms, recent studies offer preliminary evidence on these relations. Some aspects of parenting (e.g., warmth) affect the development of internalizing symptoms from childhood to early adolescence similarly across cultures, whereas other aspects of parenting (e.g., behavioral control) demonstrate more culturally specific effects on internalizing symptoms [45]. Much of this cross-national work has examined younger children (starting at age 8 years) and parenting practices relevant to such ages. The current study examined a slightly older adolescent subsample of prior cross-cultural work (e.g., [45]) while examining parenting behaviors that are particularly relevant and influential to adolescence (e.g., [41]). As such, we expected to find pancultural effects of parental monitoring and psychological control (and their interaction) on internalizing symptoms.

### Temperament X parenting interactions

There is growing interest in whether adolescent temperament and parenting interact to predict internalizing problems. Among American adolescents, Cui et al. [17] found the association between parental psychological control and adolescent depressive symptoms was stronger among adolescents with poor sadness regulation. However, Leve et al. [26] found child temperament and harsh discipline independently (not their interaction) predicted increases in internalizing problems among Americans 5–17 years old. Among Dutch preadolescents, Oldehinkel et al. [39] found youths’ frustration increased the positive association between parental overprotection and depressive symptoms. In a different study of Dutch adolescents, however, interactions between adolescent personality and parental psychological control did not predict internalizing symptoms [29]. Preliminary evidence based on constructs most closely aligned with the current study (i.e., [17]) suggests that adolescent temperament may interact with parenting to predict internalizing symptoms in the current study. Cross-cultural evidence on these interaction effects is limited, and thus exploratory in the current study.

## Method

### Participants

Participants were part of the larger study Parenting Across Cultures (PAC; e.g., [25]). We longitudinally examined 544 adolescents (T1: *M*_age_ = 12.58, SD = 0.68; 49.5% female; T2: *M*_age_ = 13.70, SD = 0.67; T3: *M*_age_ = 16.03, SD = 0.77; T4: *M*_age_ = 16.86, SD = 0.75) and their mothers (*n* = 530). Families were recruited from Medellín, Colombia; Naples and Rome, Italy; and Durham, North Carolina, United States, representing six cultural groups^2^ (i.e., Colombian, Neapolitan, Roman, African American, European American, and Latinx). Table S1 reports sample sizes for each cultural group separately for mothers and adolescents at each time-point. Adolescent participation rates were high across time (i.e., 89–98%). Table S2 summarizes maternal educational level, marital status, and number of siblings for each cultural group.

### Procedure

Following Institutional Review Board protocol, once informed consent was obtained participants were enrolled in each country until target sample sizes were reached. Participants were recruited from diverse schools with high-, middle-, and low-income families approximately matched to the socioeconomic stratification of the population of each site. Measures were administered in the predominant language of the family. We used forward and back translation to guarantee the conceptual and linguistic equivalence of instruments across languages (see [32]). Measures were administered in Spanish for Colombian families, Italian for families in Rome and Naples, and American English for African American and European American families. Latinx families were given the choice to complete measures in Spanish or English. Interviews were conducted in participants' homes or other preferred location. Each interview lasted approximately one hour. Participants were given modest financial compensation.

### Measures

#### Demographic variables

Child gender (0 = boys, 1 = girls) and family socioeconomic status (SES) at Time 1 were covariates. Mean scores of the standardized level of parental education and family income were indicators of family SES (*r* = 0.72, *p* < 0.001).

#### Negative emotionality and effortful control (T1)

Mothers completed 17 negative emotionality and 21 effortful control items on the Early Adolescent Temperament Questionnaire-Revised (EATQ-R; [13]), indicating how well statements described their child (1 “almost always untrue” to 5 “almost always true”). Negative emotionality items (e.g., Gets very irritated when someone criticizes him/her) were averaged to create a composite score (mean α across sites = 0.89). Effortful control items (e.g., Usually finishes her/his homework before it’s due) were averaged to create a composite score (mean α across sites = 0.86). Previous studies have supported the psychometric properties of this instrument in the cultural groups included in this study (e.g., [13, 19, 55]).

#### Parental monitoring (T1)

Mothers completed 10 items derived from Conger et al. [15] and Steinberg et al. [49]. For five items, mothers indicated how much they try to gain knowledge about their child’s activities and whereabouts (e.g., “How much do you try to know who your child spends time with?, 0 “I do not try” to 2 “I try a lot”). For another five items, mothers indicated how often they impose limits on their child’s activities (e.g., “How often do you set rules or limits on who your child spends time with?”; 0 “Never” to 3 “Always”). Several studies provide evidence of the reliability of the scale across the cultural groups considered in this study (e.g., [46]). All items were standardized. We created a composite variable by averaging across items (mean α across sites = 0.83).

#### Parental psychological control (T1)

Adolescents completed seven items derived from Barber [6], indicating how much their parents made decisions for them or tried to psychologically manipulate their feelings and decisions (e.g., “My parents won't let me do things with them when I do something they don't like”, 1 “Strongly disagree” to 4 “Strongly agree”). Previous studies provide evidence of the equivalence of this scale across several cultures [8, 16]. We created a composite variable by averaging across items (mean α across sites = 0.59).^3^

#### Internalizing symptoms (T2–T4)

Adolescents completed 29 items from the Youth Self-Report (YSR; [1]), referencing sadness, loneliness, withdrawal, and anxiety (e.g., “I cry a lot.”,0 “not true” to 2 “very true/often true”) during the last six months. Several studies provide evidence of the equivalence of the YSR across cultures and languages (e.g., [1]). Items were averaged to create a total score (mean α across sites and years = 0.90).

### Data analytic approach

We used Latent Growth Curve Modeling (LGCM) adjusted for unequal time points with maximum-likelihood estimation in M*Plus* 7 [35] to assess the development of internalizing symptoms in the full sample. We estimated two latent factors: (1) the intercept, representing initial levels of internalizing symptoms at T2 and (2) the slope, representing the rate of change in symptoms over time. To identify the best fitting trajectory, we tested three unconditional models: a random-intercept only, no growth model, a linear growth model; and a nonlinear growth model with no “a priori” change estimates. Because models were nested, we performed a chi-square difference test (Δχ^2^) to identify the best fitting model [22].

We then assessed possible cultural differences in the development of internalizing symptoms using multi-group analyses (e.g., [45]). We estimated an unconstrained model where no parameters estimated were constrained to be equal across groups and compared this model to a model where all structural paths were constrained to be equal across groups. If the Δχ^2^ between the constrained and unconstrained multi-group models was significant (*p* < 0.05), we examined modification indices to release paths that differed across groups [14]. Due to limited evidence on cross-cultural differences in the development of internalizing symptoms and considering the exploratory nature of the present study, we implemented a data-driven approach based on examination of modification indices to detect cross-cultural differences. The final model from these analyses was used as our baseline model when examining predictors of symptom growth.

We tested our conditional LGCM using the full sample where we considered T1 negative emotionality, effortful control, parental monitoring, psychological control, and mean-centered interactions among these variables as predictors of initial levels and change in internalizing symptoms. Adolescent gender and family SES were covariates. Significant interaction terms on the intercept and slope factor were explored post hoc by plotting values of the intercept and slope at high and low (± 1 SD) levels of temperament and parenting. Last, we ran a multi-group conditional LGCM to examine potential differences among cultural groups in how predictors and interaction terms predicted internalizing symptoms.

## Results

### Descriptive statistics and correlations

Table S3 reports means, standard deviations, skewness, and kurtosis for variables from T1 to T4 within the overall sample. Correlations among variables within the overall sample are in Table S4. Descriptive statistics and correlations separately by the cultural group are in Tables S5–S11.

### Unconditional LGCMs

The delta chi-square test (∆χ^2^[3] = 63.71, *p* < 0.001) indicated the linear change model (χ^2^[2] = 31.68, *p* < 0.001, RMSEA = 0.17 (90% CI 0.12, 0.22), CFI = 0.95, TLI = 0.93, SRMR = 0.06) fit better than the no-growth model. The linear model was similar to the non-linear growth model with no “a priori” change estimates (∆χ^2^[1] = 0.09; *p* = 0.76), indicating that in both models there was a slight linear increase in mean-level internalizing symptoms over time.

We evaluated possible differences in the development of internalizing symptoms using multi-group analyses across the six groups. The unconditional multi-group fully constrained model, where parameters were constrained to equality across the six groups, was statistically different (∆χ^2^[30] = 91.94, *p* < 0.001) from the fully unconstrained model with freely estimated parameters, suggesting significant differences across groups. The final partially constrained model fit the data well (χ^2^[37] = 68.65, *p* < 0.01, RMSEA = 0.09 (90% CI 0.06, 0.13), CFI = 0.95, TLI = 0.97, SRMR = 0.12) and was not statistically different from the fully unconstrained model (∆χ^2^[13] = 17.51, *p* = 0.17). In the final partially constrained model, eight parameters varied across groups. Variances of the slope for African Americans (*s*^2^ = 0.009, *p* < 0.001) and European Americans (*s*^2^ = 0.008, *p* < 0.001) were significantly different from the other groups (*s*^2^ = 0.006, *p* < 0.001). Furthermore, the mean slope for African Americans was negative and significant (*M* = −0.03, *p* = 0.01) whereas the slope was positive and significant for the other groups (*M* = 0.02, *p* = 0.01). The mean intercept for Latinx (*M* = 0.33, *p* < 0.001) differed significantly from other groups (*M* = 0.46, *p* < 0.001). The variance of T3 internalizing symptoms for Naples (*s*^2^ = 0.04, *p* < 0.001), African Americans (*s*^2^ = 0.05, *p* < 0.001), European Americans (*s*^2^ = 0.06, *p* < 0.001) and Colombians (*s*^2^ = 0.07, *p* < 0.001) differed significantly from other groups (*s*^2^ = 0.03, *p* < 0.001). The variance of the intercept (*s*^2^ = 0.08, *p* < 0.001) and the correlation between intercept and slope (*r* = −0.01, *p* < 0.001) were invariant across the six groups.

### Conditional LGCMs

**Fig. 1:**
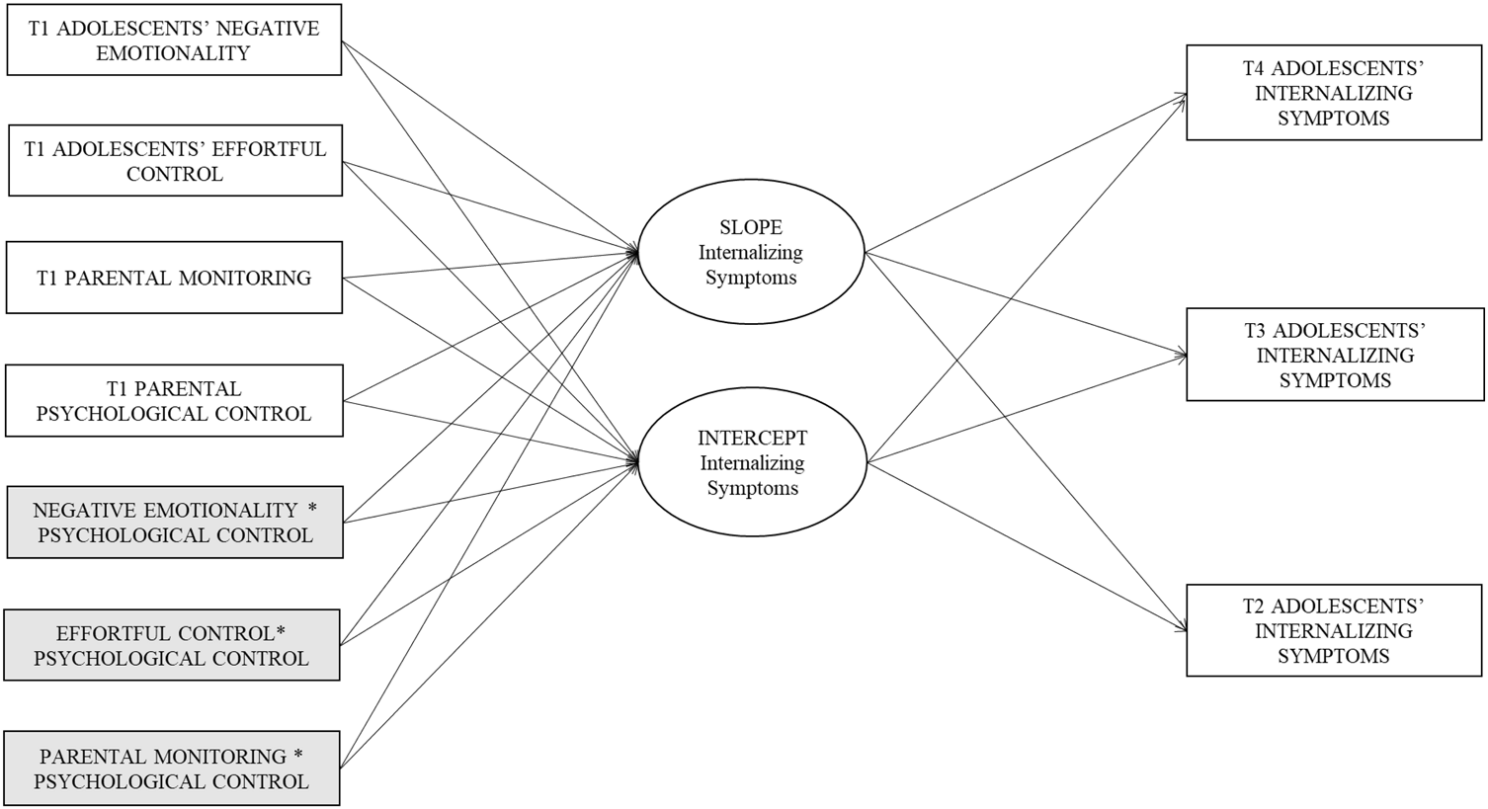
Conditional multi-group latent growth curve model for the six groups. *Note* We also estimated the correlations within the T1 predictors and the effect of covariates

To examine how well temperament and parenting predicted initial levels and growth in internalizing symptoms cross-culturally, we ran conditional multi-group LGCMs across the six groups (Fig. 1). To guarantee model parsimony [45], we excluded (a) interactions that were not significantly associated with the intercept and slope in the full sample, (b) non-significant within T1 correlations among predictors, and (c) non-significant effects of covariates on predictors and growth parameters. We preliminarily proceeded without covariate effects. We left unconstrained across groups the eight parameters that were not invariant across groups in the final unconditional partially constrained multi-group LGCM, and we examined modification indices to release paths that differed across groups. The final conditional multi-group partially constrained model (without covariate effects) fit the data well (χ^2^[229] = 270.08, *p* = 0.032, RMSEA = 0.04 (90% CI 0.01, 0.06), CFI = 0.94, TLI = 0.96, SRMR = 0.09) and was not statistically different from the fully unconstrained model (∆χ^2^[103] = 121.41, *p* = 0.10). Subsequently, we included parameters in which covariates had significant effects on predictors and growth factors. The conditional multi-group partially constrained model where the effects of covariates were constrained to equality across the six groups was statistically different (∆χ^2^[25] = 78.40, *p* < 0.001) from the correspondent model in which the effects of covariates were fully unconstrained across the six groups, suggesting some significant differences across groups in terms of covariate effects. The final conditional multi-group partially constrained model with partially constrained covariate effects across the six groups fit the data well (χ^2^[338] = 436.64, *p* < 0.001, RMSEA = 0.06 (90% CI 0.04, 0.07), CFI = 0.94, TLI = 0.93, SRMR = 0.09) and was not statistically different from the correspondent model in which the effects of covariates were fully unconstrained across the six groups (∆χ^2^[19] = 19.58, *p* = 0.42).

Overall, in this final model, we incrementally released 26 within T1 correlations among predictors, 4 effects of covariates on some predictors, and the following 5 parameters to be different across groups (1) the mean slope for Latinx (*M* = −0.05, *p* = 0.19) and for African Americans (*M* = −0.10, *p* = 0.01) differed significantly from the other cultural groups (*M* = −0.07, *p* = 0.09); (2) the variance of the intercept for Latinx (*s*^2^ = 0.04, *p* < 0.001) differed significantly from the other groups (*s*^2^ = 0.06, *p* < 0.001); (3) the relation of parental monitoring to the intercept for Latinx (*b* = 0.02, *p* = 0.44) differed from the other groups (*b* = −0.08, *p* < 0.001); (4) the relation of the interaction between effortful control and psychological control to the intercept for European Americans (*b* = −0.18, *p* = 0.01) differed from the other groups (*b* = 0.04, *p* = 0.40); and (5) the variance of T2 internalizing symptoms for African Americans (*s*^2^ = 0.29, *p* < 0.001) differed from the other groups (*s*^2^ = 0.16, *p* = 0.004).

**Fig. 2:**
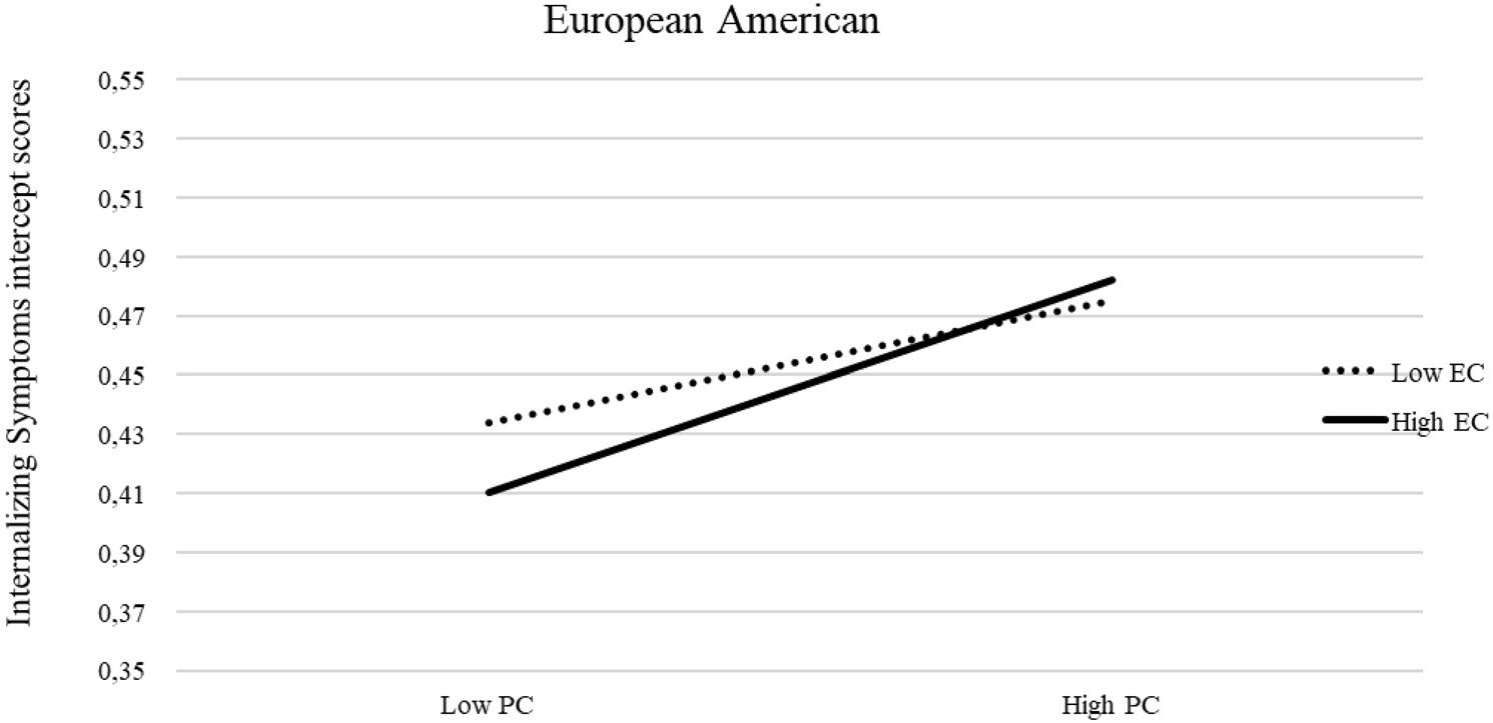
Interaction between parental psychological control and early adolescents’ effortful control in predicting the intercept of adolescents’ internalizing symptoms among European Americans. *Note* PC: parental psychological control, EC: early adolescents’ effortful control. Simple slopes are unstandardized regression coefficients

Recall that in our prior unconditional model a positive mean of the slope emerged for all groups except African Americans, for whom the mean of the slope was negative. In our final conditional multi-group partially constrained model, the inclusion of predictors and covariates affected significantly the mean of the slope. Specifically, the mean slope was no longer significant, suggesting an overall stable trajectory of internalizing symptoms for all groups except African Americans, who maintained a significant decreasing trajectory of internalizing symptoms similar to that found in the unconditional model. The details of the unstandardized estimates of invariant and variant within-time correlations among predictors and covariate effects of the final conditional multi-group partially constrained model separately by the cultural group are in tables S12-S17. The details of the unstandardized estimates of invariant and variant growth parameters and the relation of predictors to growth parameters are reported in Table 1. In terms of the relation of the predictors to the intercept of the growth curve of internalizing symptoms, T1 negative emotionality positively predicted whereas T1 effortful control negatively predicted initial levels of internalizing symptoms similarly across groups; T1 parental monitoring was negatively related to the intercept of internalizing symptoms in all groups except Latinx. T1 parental psychological control was positively related to initial levels of internalizing symptoms across groups; the interaction between effortful control and psychological control positively predicted the intercept of internalizing symptoms only among European American youths (Fig. 2). Specifically, at lower levels of psychological control, youth with lower effortful control experienced more age 14 internalizing symptoms than youth with higher effortful control; however, at higher levels of psychological control, youth with higher effortful control and youth with lower effortful control did not significantly vary from one another in their internalizing symptoms. In terms of the relation of the predictors to the slope of the growth curve of internalizing symptoms, the positive relation of effortful control to the slope was similar across groups.

**Table 1:** Unconditional and conditional multi-group latent growth curve models

	Estimate^a^	SE	*p* value
Unconditional model			
Intercept with slope^b^	−0.01	0.00	< 0.001
Intercept variance	0.07	0.01	< 0.001
Intercept mean	0.46; 0.33^c^	0.01; 0.03^c^	< 0.001; < 0.001^c^
Slope variance	0.006; 0.009^d^; 0.008^e^	0.00; 0.00^d^; 0.00^e^	< 0.001; <0 .001^d^; <0 .001^e^
Slope mean	0.02; −0.03^d^	0.00; 0.01^d^	0.01; 0.01^d^
Conditional model			
Intercept with slope^b^	−0.01	0.00	< 0.001
Intercept variance	0.06; 0.04^c^	0.01; 0.01^c^	< 0.001; < 0.001^c^
Intercept mean	0.25; 0.11^c^	0.12; 0.13^c^	0.04; 0.39^c^
Slope variance	0.005; 0.008^d^; 0.007^e^;	0.00; 0.00^d^; 00.00^e^	< 0.001; < 0.001; < 0.001
Slope mean	−0.07; −0.05^c^; −0.10^d^	0.04; 0.04^c^; 0.04^d^	0.09; 0.19^c^; 0.01^d^
Predictors of intercept			
Negative emotionality	0.06	0.02	<0 .001
Effortful control	−0.05	0.02	0.03
Parental monitoring	−0.08; 0.02^c^	0.02; 0.03^c^	0.002; 0.44^c^
Psychological control	0.06	0.02	0.008
Negative emotionality × psychological control	−0.03	0.04	0.40
Effortful control × psychological control	0.04; 0.18^e^	0.04; .06^e^	0.40; 0.01^e^
Parental monitoring × psychological control	0.05	0.04	0.28
Predictors of linear slope			
Negative emotionality	0.01	0.01	0.06
Effortful control	0.01	0.01	0.02
Parental monitoring	−0.00	0.00	0.59
Psychological control	−0.00	0.01	0.92
Negative emotionality × psychological control	−0.02	0.01	0.10
Effortful control × psychological control	−0.02	0.01	0.19
Parental monitoring × psychological control	0.01	0.01	0.86

Superscripts c through e indicate parameters for which the equality constraint was lifted in one cultural group in comparison to the other ones

^a^Estimates are unstandardized betas unless otherwise indicated

^b^estimate is a correlation coefficient

^c^Latinx

^d^African American

^e^European American

## Discussion

Early adolescent temperament and parenting behaviors predict the development of internalizing symptoms during adolescence, yet cross-national longitudinal evidence on these relations is limited. Furthermore, the limited empirical investigations in this area have only rarely considered the joint and interactive relation of temperament and parenting to the development of internalizing symptoms using a cross-cultural lens. Therefore, the present study was exploratory in its aims and used a data-driven approach to partially fill this gap by investigating (1) the longitudinal development of internalizing symptoms among normative youth from six cultural groups in three countries (Colombian, Neapolitan Italian, Roman Italian, African American, European American, and Latinx), (2) early-adolescent temperament, parenting behaviors, and their interactions as predictors of initial levels and growth of internalizing symptoms, and (3) cross-cultural commonalities (or specificities) in our findings.

We found that age-14 internalizing symptoms were similar for five of our six cultural groups. Latinx youth had lower internalizing symptoms at age 14. This is consistent with earlier work with partially the same sample of youth [45] where internalizing symptoms from ages 10–12 decreased for Latinx youth and either increased or remained stable for other American, Colombian, and Italian youth. Thus, internalizing symptoms for Latinx youth had a lower starting point than other youth. Our samples were a subset of those examined in Rothenberg et al. [45], and our findings are consistent with their findings. We found an average increase in internalizing symptoms during adolescence in five of our six cultural groups. Adolescents frequently manage great changes in their social and emotional worlds, with increased needs for autonomy not always being satisfied, increased parent–child conflicts related to their emerging need for greater autonomy, substantial biological and hormonal changes often associated with decreases in self-esteem, and ongoing challenges defining identity [48]. These factors as well as others might account for increases in normative experiences of internalizing symptoms during adolescence.

African American adolescents experienced an average decline in internalizing symptoms. This finding expands earlier work suggesting that while internalizing symptoms among African American youth may remain level during late childhood and early adolescence [45], during early- to mid-adolescence African American youth may begin experiencing decreases in their levels of internalizing symptoms. Previous cross-sectional studies suggest high comorbidity in internalizing and externalizing problems among African American youth [27]. African American adolescents may express anxiety, withdrawal, and depression jointly with externalizing symptoms (e.g., anger and irritability), and less as purely internalizing symptoms (e.g., [2]). Thus, our measurement of just internalizing symptoms may not fully be the best representation of African American youths’ internalizing symptoms. Future longitudinal work examining the possible joint manifestation of both internalizing and externalizing symptoms among African American youth is needed.

As stated before, in our model without predictors (unconditional model) we found that internalizing symptoms increased over time during adolescence for all groups except African Americans in the United States (for whom internalizing symptoms decreased over time). After including adolescent gender, family SES, youth temperament, and parenting as predictors of growth in internalizing symptoms (conditional model), the slope of internalizing symptoms changed from significantly positive to non-significant (i.e., it was stable over time) for Colombians, Italians, European Americans, and Latinx while it remained significantly negative for African Americans. In addition, we found that early adolescent temperament and parenting behaviors at age 13 predicted initial levels of internalizing symptoms at age 14 and change in internalizing symptoms from ages 14 to 17. Higher parental monitoring at age 13 was associated with lower levels of internalizing symptoms at age 14 (except for Latinx youth), which is consistent with some previous findings (e.g., [24]. High parental monitoring may protect against the development of internalizing symptoms by improving closeness and disclosure in the parent–child relationship and through parents’ abilities to monitor their adolescent’s emotional and social life in non-intrusive ways [47]. Consistent with previous studies (e.g., [17], 39, 50), we found a significant positive association between parental psychological control and initial levels of internalizing symptoms measured one year later. Parents who are psychologically controlling invalidate adolescents’ thoughts, feelings, and autonomy [24], which may increase their internalizing symptoms. Prior cross-sectional evidence suggested that psychological control may interact with parental monitoring to predict internalizing symptoms [42], however, we did not find such an interactive effect. Finally, as expected, the main effects of parental monitoring and psychological control were pancultural, highlighting the generalizable influence such parenting behaviors might have on internalizing symptoms during adolescence.

Regarding temperament, across all cultural groups we found a significant positive relation between age 13 negative emotionality and age 14 internalizing symptoms. The notable changes adolescents experience in their emotional lives could make them more vulnerable to internalizing symptoms when those emotional experiences are negative. Furthermore, across all cultural groups, we found a significant negative relation between age 13 effortful control and age 14 internalizing symptoms (the intercept effect) as well as a significant positive relation between age-13 effortful control and growth in internalizing symptoms over time (the slope effect). The pancultural effect of greater age 13 effortful control predicting fewer age 14 internalizing symptoms (the intercept effect) was much larger (*B* = −0.05) than the relatively small pancultural positive association between greater age 13 effortful control and greater increases in internalizing symptoms across ages 14–16 (i.e., the slope effect; *B* = 0.01). The cross-cultural protective effect of age 13 effortful control on internalizing symptoms one year later is consequently not completely negated by the increases in internalizing symptoms through age 16 (the last age studied here) associated with higher effortful control. Therefore, overall, effortful control still exhibited a cross-culturally protective effect against internalizing symptoms in the current sample, even though that effect faded slightly over time.

Some prior work [38] suggested that negative emotionality might interact with effortful control to predict internalizing symptoms, however, we did not find such an interactive effect. Nonetheless, the main effects of adolescent temperament on internalizing symptoms were pancultural, which expands the limited, yet growing, evidence on the long-term influence temperament might have on adolescent adjustment across cultures. Future cross-national investigations of temperament and psychological adjustment are essential for uncovering additional ways temperament might influence dimensions of psychological adjustment beyond internalizing symptoms.

The interaction between effortful control and parental psychological control predicted the intercept of internalizing symptoms only among European Americans. At lower levels of parental psychological control, adolescents with lower effortful control reported notably more internalizing symptoms (one year later) than adolescents with higher effortful control. However, at higher levels of parental psychological control, youth with higher effortful control and youth with lower effortful control did not significantly vary from one another in their reported levels of internalizing symptoms one year later. This finding suggests that the effects of psychological control are notably detrimental to youth’s psychological adjustment in that they can “overwhelm” even the protective effects offered by high levels of effortful control. Our findings in this regard were specific to European Americans, which adds new evidence on cultural differences in the interaction between parenting and temperament during adolescence, further demonstrating the importance of future cross-national investigations of predictors of internalizing symptoms.

This study is one of few examining the development of internalizing symptoms during adolescence while examining potential cultural differences. To our knowledge, it is the only longitudinal study considering the unique and interactive effects of temperament and parenting predicting internalizing symptoms during adolescence. Nonetheless, this study had limitations. We did not investigate other parenting variables, such as parental warmth, acceptance/rejection, and punitive parenting, known to influence adolescents’ internalizing symptoms. We also did not investigate the measurement invariance of the scales across the six cultural groups. However, as previously reported, several studies suggest invariance of the measures among the cultural groups used in the present study. Furthermore, we cannot exclude more complex developmental trajectories of internalizing symptoms requiring more than three data points. The relatively small sample sizes within the six cultural groups are another limitation of this study. However, in accordance with previous studies (e.g., [30]) establishing a minimum desirable sample size (i.e., subjects-to-variables (STV) ratio of 3:1), the within-group sample sizes of this study are within the range of acceptable minimum sample sizes (smallest STV ratio of 4.75:1). Lastly, our data were correlational, prohibiting causal conclusions. Nonetheless, the main effects of our predictors remained invariant across cultures providing evidence on the generalizability of our findings across Colombia, Italy, and the US, and highlighting the importance of research and prevention targeting adolescents’ self-regulation and negative emotionality, as well as parenting behaviors, to fully comprehend internalizing symptoms around the world.

## Supplementary Information

Below is the link to the electronic supplementary material.

Supplementary file1 (DOCX 76 KB)

## Footnotes

1 Throughout this manuscript, we use the term Latinx instead of Hispanic because Latinx is a more inclusive term and reflects current terminology in practice within the United States.

2 Our selection of and distinctions among the six cultural groups in this study are based on existing evidence that these six groups vary in meaningful ways from each other on a variety of dimensions, especially those pertaining to parent–child relationships and parenting variables of particular relevance to this study. For instance, Italian family identities are characterized by distinct regional dialects and different cultural traditions that indicate a specific regional (as opposed to national) identity. Families from Rome (which is centrally located) have similar characteristics to families in the regions of Northern Italy which differ from families in regions located in Southern Italy (e.g., Naples). Cultural and economic differences between Northern/Central and Southern regions of Italy are reflected in differences in parenting attitudes and styles of mothers and fathers [9]. Within the United States, extensive empirical evidence indicates that parenting practices and their consequent influences on youth development vary by ethnicity (e.g., Gunnoe et al., 1997,[23]. Therefore, a substantial body of cross-cultural parenting research has treated data from Rome and Naples separately as well as data from European Americans, African Americans, and Latinx families separately (e.g., [25],[45].

3 Item-total correlations ranged from .19 to .38. Based on factor analysis, all indicators loaded on the factor of psychological control > .30, except for one item (.24). Overall, items appeared at least modestly interrelated.

## References

[1] Achenbach TM (1994) Integrative guide for the 1991 CBCL/4-18, YSR, and TRF profiles. Department of Psychiatry, University of Vermont

[2] Anderson ER, Mayes LC (2010). Race/ethnicity and internalizing disorders in youth: a review. Clin Psychol Rev.

[3] Asendorpf JB, van Aken MAG (1999). Resilient, overcontrolled, and undercontrolled personality prototypes in childhood: replicability, predictive power, and the trait-type issue. J Pers Soc Psychol.

[4] Avenevoli S, Swendsen J, He JP, Burstein M, Merikangas KR (2015). Major depression in the national comorbidity survey–adolescent supplement: prevalence, correlates, and treatment. J Am Acad Child Adolesc Psychiatry.

[5] Balan R, Dobrean A, Roman GD, Balazsi R (2017). Indirect effects of parenting practices on internalizing problems among adolescents: the role of expressive suppression. J Child Fam Stud.

[6] Barber BK (1996). Parental psychological control: revisiting a neglected construct. Child Dev.

[7] Barber BK, Bean RL, Erickson LD, Barber BK (2002). Expanding the study and understanding of parental psychological control. Intrusive parenting: how psychological control affects children and adolescents.

[8] Barber BK, Stolz HE, Olsen JA, Collins WA, Burchinal M (2005). Parental support, psychological control, and behavioral control: assessing relevance across time, culture, and method. Monogr Soc Re Child Dev.

[9] Bombi AS, Pastorelli C, Bacchini D, Di Giunta L, Miranda MC, Zelli A (2011). Attributions and attitudes of mothers and fathers in Italy. Parenting.

[10] Bongers IL, Koot HM, Van der Ende J, Verhulst FC (2003). The normative development of child and adolescent problem behavior. J Abnorm Psychol.

[11] Bowers EP, Gestsdottir S, Geldhof GJ, Nikitin J, von Eye A, Lerner RM (2011). Developmental trajectories of intentional self regulation in adolescence: the role of parenting and implications for positive and problematic outcomes among diverse youth. J Adolesc.

[12] Cai T, Tu KM (2020). Linking parental monitoring and psychological control with internalizing symptoms in early adolescence: the moderating role of vagal tone. J Abnorm Child Psychol.

[13] Capaldi DM, Rothbart MK (1992). Development and validation of an early adolescent temperament measure. J Early Adolesc.

[14] Cheung GW, Rensvold RB (2002). Evaluating goodness-of-fit indexes for testing measurement invariance. Struct Equ Model.

[15] Conger RD, Ge X, Elder GH, Lorenz FO, Simons RL (1994). Economic stress, coercive family processes, and developmental problems of adolescents. Child Dev.

[16] Costa S, Soenens B, Gugliandolo MC, Cuzzocrea F, Larcan R (2015). The mediating role of experiences of need satisfaction in associations between parental psychological control and internalizing problems: a study among Italian college students. J Child Fam Stud.

[17] Cui L, Morris AS, Criss MM, Houltberg BJ, Silk JS (2014). Parental psychological control and adolescent adjustment: the role of adolescent emotion regulation. Parenting.

[18] Eisenberg N, Sadovsky A, Spinrad TL, Fabes RA, Losoya SH, Valiente C (2005). The relations of problem behavior status to children's negative emotionality, effortful control, and impulsivity: concurrent relations and prediction of change. Dev Psychol.

[19] Esposito C, Bacchini D, Eisenberg N, Affuso G (2017). Effortful control, exposure to community violence, and aggressive behavior: exploring cross-lagged relations in adolescence. Aggress Behav.

[20] Galambos NL, Barker ET, Almeida DM (2003). Parents do matter: trajectories of change in externalizing and internalizing problems in early adolescence. Child Dev.

[21] Gunnoe ML, Mariner CL (1997). Toward a developmental-contextual model of the effects of parental spanking on children’s aggression. Arch Pediatr Adolesc Med.

[22] Kline RB, Thompson B, Subotnik RE (2010). Promise and pitfalls of structural equation modeling in gifted research. Methodologies for conducting research on giftedness.

[23] Lansford JE, Deater-Deckard K, Dodge KA, Bates JE, Pettit GS (2004). Ethnic differences in the link between physical discipline and later adolescent externalizing behaviors. J Child Psychol Psychiatry.

[24] Lansford JE, Laird RD, Pettit GS, Bates JE, Dodge KA (2014). Mothers’ and fathers’ autonomy-relevant parenting: longitudinal links with adolescents’ externalizing and internalizing behavior. J Youth Adolesc.

[25] Lansford JE, Rothenberg WA, Jensen TM, Lippold MA, Bacchini D, Bornstein H (2018). Bidirectional relations between parenting and behavior problems from age 8 to 13 in nine countries. J Res Adolesc.

[26] Leve LD, Kim HK, Pears KC (2005). Childhood temperament and family environment as predictors of internalizing and externalizing trajectories from ages 5 to 17. J Abnorm Child Psychol.

[27] Liu J, Mustanski B, Dick D, Bolland J, Kertes DA (2017). Risk and protective factors for comorbid internalizing and externalizing problems among economically disadvantaged African American youth. Dev Psychopathol.

[28] Loukas A, Prelow HM (2014). Externalizing and internalizing problems in low-income Latino early adolescents: risk, resource, and protective factors. J Early Adolesc.

[29] Mabbe E, Vansteenkiste M, Brenning K, De Pauw S, Beyers W, Soenens B (2019). The moderating role of adolescent personality in associations between psychologically controlling parenting and problem behaviors: a longitudinal examination at the level of within-person change. Dev Psychol.

[30] MacCallum RC, Widaman KF, Zhang S, Hong S (1999). Sample size in factor analysis. Psychol Methods.

[31] Maciejewski DF, van Lier PA, Branje SJ, Meeus WH, Koot HM (2017). A daily diary study on adolescent emotional experiences: measurement invariance and developmental trajectories. Psychol Assess.

[32] Maxwell B (1996) Translation and cultural adaptation of the survey instruments. In: Martin MO, Kelly DL (eds) Third International Mathematics and Science Study (TIMSS) technical report, vol 1. Design and development. Boston College, Chestnut Hill. http://timss.bc.edu/timss1995i/TIMSSPDF/TRall.pdf

[33] Moriya J, Tanno Y (2008). Relationships between negative emotionality and attentional control in effortful control. Personal Individ Differ.

[34] Muhtadie L, Zhou Q, Eisenberg N, Wang Y (2013). Predicting internalizing problems in Chinese children: the unique and interactive effects of parenting and child temperament. Dev Psychopathol.

[35] Muthén LK, Muthén BO (2012). Version 7 Mplus user’s guide.

[36] Nigg JT (2006). Temperament and developmental psychopathology. J Child Psychol Psychiatry.

[37] Oldehinkel AJ, Hartman CA, De Winter AF, Veenstra R, Ormel J (2004). Temperament profiles associated with internalizing and externalizing problems in preadolescence. Dev Psychopathol.

[38] Oldehinkel AJ, Hartman CA, Ferdinand RF, Verhulst FC, Ormel J (2007). Effortful control as modifier of the association between negative emotionality and adolescents' mental health problems. Dev Psychopathol.

[39] Oldehinkel AJ, Veenstra R, Ormel J, De Winter AF, Verhulst FC (2006). Temperament, parenting, and depressive symptoms in a population sample of preadolescents. J Child Psychol Psychiatry.

[40] Oliva A, Arranz E, Parra A, Olabarrieta F (2014). Family structure and child adjustment in Spain. J Child Fam Stud.

[41] Pinquart M (2017). Associations of parenting dimensions and styles with internalizing symptoms in children and adolescents: a meta-analysis. Marriage Fam Rev.

[42] Rodríguez-Meirinhos A, Vansteenkiste M, Soenens B, Oliva A, Brenning K, Antolín-Suárez L (2020). When is parental monitoring effective? A person-centered analysis of the role of autonomy-supportive and psychologically controlling parenting in referred and non-referred adolescents. J Youth Adolesc.

[43] Rothbart MK, Bates JE, Damon W, Eisenberg N (2006). Temperament. Handbook of child psychology.

[44] Rothbart MK, Ellis LK, Posner MI, Baumeister RF, Vohs KD (2004). Temperament and self-regulation. Handbook of self-regulation: research, theory, and applications.

[45] Rothenberg WA, Lansford JE, Bornstein MH, Chang L, Deater-Deckard K, Di Giunta L (2020). Effects of parent warmth and behavioral control on adolescent externalizing and internalizing trajectories across cultures. J Res Adolesc.

[46] Skinner AT, Bacchini D, Lansford JE, Godwin JW, Sorbring E, Tapanya S (2014). Neighborhood danger, parental monitoring, harsh parenting, and child aggression in nine countries. Societies.

[47] Stattin H, Kerr M (2000). Parental monitoring: a reinterpretation. Child Dev.

[48] Steinberg L, Morris AS (2001). Adolescent development. Annu Rev Psychol.

[49] Steinberg L, Lamborn SD, Dornbusch SM, Darling N (1992). Impact of parenting practices on adolescent achievement: Authoritative parenting, school involvement, and encouragement to succeed. Child Dev.

[50] Symeou M, Georgiou S (2017). Externalizing and internalizing behaviours in adolescence, and the importance of parental behavioural and psychological control practices. J Adolesc.

[51] Taylor RD, Lopez EI, Budescu M, McGill RK (2012). Parenting practices and adolescent internalizing and externalizing problems: moderating effects of socially demanding kin relations. J Child Fam Stud.

[52] Twenge JM, Nolen-Hoeksema S (2002). Age, gender, race, socioeconomic status, and birth cohort difference on the children's depression inventory: a meta-analysis. J Abnorm Psychol.

[53] UNICEF (2017). Standards for ECD parenting programmes in low and middle income countries.

[54] Van Loon LMA, Van De Ven MOM, Van Doesum KTM, Hosman CMH, Witteman CLM (2015). Factors promoting mental health of adolescents who have a parent with mental illness: a longitudinal study. Child Youth Care Forum.

[55] Viñas Poch F, González Carrasco M, Gras Pérez E, Jane Ballabriga C, Casas Aznar F (2015). Psychometric properties of the EATQ-R among a sample of Catalan-speaking Spanish adolescents. Univ Psychol.

[56] Wang F, Cox MJ, Mills-Koonce R, Snyder P (2015). Parental behaviors and beliefs, child temperament, and attachment disorganization. Fam Relat.

[57] Zahn-Waxler C, Klimes-Dougan B, Slattery MJ (2000). Internalizing problems of childhood and adolescence: prospects, pitfalls, and progress in understanding the development of anxiety and depression. Dev Psychopathol.

