# Supplementary material for: Development of internalizing symptoms during adolescence in three countries: the role of temperament and parenting behaviors
Source: Eur Child Adolesc Psychiatry. 2021 Feb 6;31(6):947–57. doi: 10.1007/s00787-021-01725-6 (PMC8610087; doi:10.1007/s00787-021-01725-6)
Supplement: Supplementary file 1 — Supplementary file1 (DOCX 76 KB) [file 787_2021_1725_MOESM1_ESM.docx]

- Supplemental Material

Table S1

*Sample sizes differentiated by site, time, and reporter.*

|  |  | **Time 1** | **Time 2** | **Time 3** | **Time 4** |
| --- | --- | --- | --- | --- | --- |
| **Colombia** |  | Adolescents *n=*88  Mothers *n=*87 | *n*=83 | *n*=76 | *n*=77 |
| **Italy** | **Naples** | Adolescents *n=*90  Mothers *n=*90 | *n*=88 | *n*=83 | *n*=85 |
|  | **Rome** | Adolescents *n=*100  Mothers *n=*102 | *n*=96 | *n*=100 | *n*=99 |
| **United States** | **African American** | Adolescents *n=*92  Mothers *n=*87 | *n*=88 | *n*=86 | *n*=85 |
|  | **European American** | Adolescents *n=*97  Mothers *n=*94 | *n*=92 | *n*=88 | *n*=88 |
|  | **Latinx** | Adolescents *n=*77  Mothers *n=*70 | *n*=71 | *n*=57 | *n*=60 |

*Note*: Only adolescents reported at Time 2 through Time 4.

Table S2

*Time 1 Sociodemographic Variables*

|  | **Colombia** | | **Italy** | | | | **United States** | | | | | | |  |
| --- | --- | --- | --- | --- | --- | --- | --- | --- | --- | --- | --- | --- | --- | --- |
|  |  |  | **Rome** | | **Naples** | | **European**  **American** | | **African**  **American** | | **Latinx** | |  | |
|  | **M** | **SD** | **M** | **SD** | **M** | **SD** | **M** | **SD** | **M** | **SD** | **M** | **SD** | |  |
| **Maternal years of education-Time 1** | 10.26^a^ | 5.46 | 14.10^b^ | 4.04 | 10.34^a^ | 4.45 | 16.88^c^ | 2.75 | 13.58^b^ | 2.36 | 10.41^a^ | 4.16 | | F(5, 536)=43.22** |
|  |  |  |  |  |  |  |  |  |  |  |  |  | |  |
| **Marital status (%) – Time 1** |  |  |  |  |  |  |  |  |  |  |  |  | | F(5, 521)=17.87** |
| **Married** | 69.3 |  | 75.5 |  | 83 |  | 81.4 |  | 37.2 |  | 55.7 |  | |  |
| **Remarried** | 0 |  | 0 |  | 2.2 |  | 2.1 |  | 1.2 |  | 2.9 |  | |  |
| **Divorced** | 0 |  | 1 |  | 1.1 |  | 7.2 |  | 9.3 |  | 7.1 |  | |  |
| **Separated** | 3.4 |  | 11.1 |  | 6.8 |  | 2.1 |  | 7 |  | 7.1 |  | |  |
| **Widowed** | 0 |  | 2 |  | 2.3 |  | 2.1 |  | 1.2 |  | 0 |  | |  |
| **Cohabiting** | 22.7 |  | 3.1 |  | 2.3 |  | 2.1 |  | 7 |  | 18.6 |  | |  |
| **Never married** | 4.5 |  | 7.1 |  | 2.3 |  | 3.1 |  | 37.2 |  | 8.6 |  | |  |
| **Number of siblings** | 2.10^a^ | 1.00 | 1.95^a^ | .80 | 2.00^a^ | .71 | 2.29^ab^ | .97 | 2.25^ab^ | 1.27 | 2.57^b^ | 1.31 | | F(5, 527)=3.96** |

*Note*. *=*p*<0.05; **=*p*<0.01. Superscripts indicate results of the Tukey Post-Hoc test. The groups with the same letter are statistically equal and different from the groups with different letters.

Table S3

*Descriptive statistics for study variables within the total sample.*

| **Time Variable (Reporter)** | **Mean** | **SD** | **Skewness** | **Kurtosis** |
| --- | --- | --- | --- | --- |
| T1 Adolescent negative emotionality (mother) | 2.40 | .74 | .45 | -.27 |
| T1 Adolescent effortful control (mother) | 3.48 | .64 | -.31 | -.16 |
| T1 Parental monitoring (mother) | -.00 | .63 | -1.38 | 2.27 |
| T1 Parental psychological control (adolescent) | 2.43 | .51 | .26 | -.00 |
| T2 Internalizing symptoms (adolescent) | .44 | .30 | .86 | .75 |
| T3 Internalizing symptoms (adolescent) | .51 | .36 | .97 | .80 |
| T4 Internalizing symptoms (adolescent) | .47 | .31 | .72 | .19 |

Table S4

*Correlations among study variables for the total sample (observed scores).*

| Variable (reporter) | (1) | (2) | (3) | (4) | (5) | (6) | (7) | (8) | (9) |
| --- | --- | --- | --- | --- | --- | --- | --- | --- | --- |
| (1) Adolescent gender | - |  |  |  |  |  |  |  |  |
| (2) Family socioeconomic status (SES) | -.04 | - |  |  |  |  |  |  |  |
| (3) T1 Adolescent negative emotionality (mother) | .04 | -.27** | - |  |  |  |  |  |  |
| (4) T1 Adolescent effortful control (mother) | .09* | .14** | -.47** | - |  |  |  |  |  |
| (5) T1 Parental monitoring (mother) | -.07 | -.06 | .05 | .14** | - |  |  |  |  |
| (6) T1 Parental psychological control (adolescent) | -.09* | -.18** | .14** | -.19** | .07 | - |  |  |  |
| (7) T2 Internalizing symptoms (adolescent) | .27** | .04 | .21** | -.14** | -.10* | -.07 | - |  |  |
| (8) T3 Internalizing symptoms (adolescent) | .40** | .03 | .19** | -.08 | -.14** | .03 | .62** | - |  |
| (9) T4 Internalizing symptoms (adolescent) | .32** | -.03 | .24** | -.10* | -.17** | .07 | .57** | .74** | - |

*Note*: Observed scores for adolescent internalizing symptoms were computed at T2, T3, and T4. Adolescent gender was coded 0=boys, 1=girls. ** = *p* < .01; * = *p* < .05.

Table S5

*Descriptive statistics for study variables reported separately by cultural group.*

|  | **Colombia** | | **Italy** | | | | **United States** | | | | | |
| --- | --- | --- | --- | --- | --- | --- | --- | --- | --- | --- | --- | --- |
|  |  | | **Naples** | | **Rome** | | **African American** | | **European American** | | **Latinx** | |
| **Time Variable (reporter)** | **Mean** | **SD** | **Mean** | **SD** | **Mean** | **SD** | **Mean** | **SD** | **Mean** | **SD** | **Mean** | **SD** |
| T1 Adolescent negative emotionality (mother) | 2.51 | .87 | 2.60 | .77 | 2.38 | .59 | 2.21 | .59 | 2.35 | .71 | 2.31 | .81 |
| T1 Adolescent effortful control (mother) | 3.54 | .73 | 3.39 | .63 | 3.45 | .54 | 3.40 | .54 | 3.56 | .67 | 3.58 | .61 |
| T1 Parental monitoring (mother) | .01 | .78 | -.14 | .61 | -.17 | .55 | .38 | .55 | -.00 | .43 | -.06 | .87 |
| T1 Parental psychological control (adolescent) | 2.41 | .53 | 2.48 | .52 | 2.37 | .50 | 2.49 | .50 | 2.27 | .51 | 2.60 | .41 |
| T2 Internalizing symptoms (adolescent) | .49 | .30 | .46 | .29 | .46 | .27 | .41 | .27 | .46 | .33 | .32 | .23 |
| T3 Internalizing symptoms (adolescent) | .61 | .41 | .54 | .34 | .53 | .31 | .53 | .31 | .56 | .39 | .40 | .29 |
| T4 Internalizing symptoms (adolescent) | .55 | .30 | .50 | .27 | .50 | .30 | .50 | .30 | .49 | .35 | .40 | .27 |

Table S6

*Correlations among study variables for Colombia.*

| Variable (reporter) | (1) | (2) | (3) | (4) | (5) | (6) | (7) | (8) | (9) |
| --- | --- | --- | --- | --- | --- | --- | --- | --- | --- |
| (1) Adolescent gender | - |  |  |  |  |  |  |  |  |
| (2) Family socioeconomic status (SES) | .03 | - |  |  |  |  |  |  |  |
| (3) T1 Adolescent negative emotionality (mother) | .08 | -.61** | - |  |  |  |  |  |  |
| (4) T1 Adolescent effortful control (mother) | -.09 | .29** | -.58** | - |  |  |  |  |  |
| (5) T1 Parental monitoring (mother) | -.08 | -.33** | .27* | -.24* | - |  |  |  |  |
| (6) T1 Parental psychological control (adolescent) | -.14 | .02 | .05 | -.07 | .06 | - |  |  |  |
| (7) T2 Internalizing symptoms (adolescent) | .39** | -.07 | .17 | -.15 | -.08 | .11 | - |  |  |
| (8) T3 Internalizing symptoms (adolescent) | .56** | -.11 | .15 | -.18 | -.06 | .14 | .65** | - |  |
| (9) T4 Internalizing symptoms (adolescent) | .42** | -.08 | .26* | -.16 | -.06 | .19 | .62** | .74** | - |

*Note*: Observed scores for adolescent internalizing symptoms were computed at T2, T3, and T4. Adolescent gender was coded 0=boys, 1=girls. ** = *p* < .01; * = *p* < .05.

Table S7

*Correlations among study variables for Italy-Naples.*

|  | (1) | (2) | (3) | (4) | (5) | (6) | (7) | (8) | (9) |
| --- | --- | --- | --- | --- | --- | --- | --- | --- | --- |
| (1) Adolescent gender | - |  |  |  |  |  |  |  |  |
| (2) Family socioeconomic status (SES) | .09 | - |  |  |  |  |  |  |  |
| (3) T1 Adolescent negative emotionality (mother) | -.14 | -.39** | - |  |  |  |  |  |  |
| (4) T1 Adolescent effortful control (mother) | .21* | .17 | -.45** | - |  |  |  |  |  |
| (5) T1 Parental monitoring (mother) | .08 | .05 | .01 | .04 | - |  |  |  |  |
| (6) T1 Parental psychological control (adolescent) | -.09 | -.45** | -.42** | -.25* | .27* | - |  |  |  |
| (7) T2 Internalizing symptoms (adolescent) | .33** | .01 | .06 | -.21 | -.21 | .00 | - |  |  |
| (8) T3 Internalizing symptoms (adolescent) | .39** | .07 | .05 | -.03 | -.25* | .06 | .66** | - |  |
| (9) T4 Internalizing symptoms (adolescent) | .31** | -.05 | .11 | -.00 | -.32** | .02 | .68** | .84** | - |

*Note*: Observed scores for adolescent internalizing symptoms were computed at T2, T3, and T4. Adolescent gender was coded 0=boys, 1=girls. ** = *p* < .01; * = *p* < .05.

Table S8

*Correlations among study variables for Italy- Rome.*

|  | (1) | (2) | (3) | (4) | (5) | (6) | (7) | (8) | (9) |
| --- | --- | --- | --- | --- | --- | --- | --- | --- | --- |
| (1) Adolescent gender | - |  |  |  |  |  |  |  |  |
| (2) Family socioeconomic status (SES) | -.08 | - |  |  |  |  |  |  |  |
| (3) T1 Adolescent negative emotionality (mother) | .10 | -.06 | - |  |  |  |  |  |  |
| (4) T1 Adolescent effortful control (mother) | .06 | .14 | -.31** | - |  |  |  |  |  |
| (5) T1 Parental monitoring (mother) | -.03 | -.16 | .04 | -.05 | - |  |  |  |  |
| (6) T1 Parental psychological control (adolescent) | -.09* | -.16 | .08 | -.21** | .10 | - |  |  |  |
| (7) T2 Internalizing symptoms (adolescent) | .36** | .06 | .28** | -.19 | -.23* | .15 | - |  |  |
| (8) T3 Internalizing symptoms (adolescent) | .44** | .00 | .26** | -.21* | -.18 | .13 | .63** | - |  |
| (9) T4 Internalizing symptoms (adolescent) | .46** | -.02 | .18 | -.20* | -.12* | .17 | .59** | .75** | - |

*Note*: Observed scores for adolescent internalizing symptoms were computed at T2, T3, and T4. Adolescent gender was coded 0=boys, 1=girls. ** = *p* < .01; * = *p* < .05.

Table S9

*Correlations among study variables for United States-African American.*

|  | (1) | (2) | (3) | (4) | (5) | (6) | (7) | (8) | (9) |
| --- | --- | --- | --- | --- | --- | --- | --- | --- | --- |
| (1) Adolescent gender | - |  |  |  |  |  |  |  |  |
| (2) Family socioeconomic status (SES) | .01 | - |  |  |  |  |  |  |  |
| (3) T1 Adolescent negative emotionality (mother) | .16 | -.15 | - |  |  |  |  |  |  |
| (4) T1 Adolescent effortful control (mother) | .10 | .23** | -.49** | - |  |  |  |  |  |
| (5) T1 Parental monitoring (mother) | -.11 | -.27** | .03 | -.01 | - |  |  |  |  |
| (6) T1 Parental psychological control (adolescent) | -.12 | -.00 | .03 | -.09 | .10 | - |  |  |  |
| (7) T2 Internalizing symptoms (adolescent) | .21 | -.08 | .22** | -.12 | -.01 | .19 | - |  |  |
| (8) T3 Internalizing symptoms (adolescent) | .29** | .08 | .12 | -.03 | -.06 | .04 | .45** | - |  |
| (9) T4 Internalizing symptoms (adolescent) | .32** | .01 | .17 | .02 | -.15 | .04 | .41** | .68** | - |

*Note*: Observed scores for adolescent internalizing symptoms were computed at T2, T3, and T4. Adolescent gender was coded 0=boys, 1=girls. ** = *p* < .01; * = *p* < .05.

Table S10

*Correlations among study variables for United States-European American.*

|  | (1) | (2) | (3) | (4) | (5) | (6) | (7) | (8) | (9) |
| --- | --- | --- | --- | --- | --- | --- | --- | --- | --- |
| (1) Adolescent gender | - |  |  |  |  |  |  |  |  |
| (2) Family socioeconomic status (SES) | -.02 | - |  |  |  |  |  |  |  |
| (3) T1 Adolescent negative emotionality (mother) | .04 | .07 | - |  |  |  |  |  |  |
| (4) T1 Adolescent effortful control (mother) | .25* | .10 | -.43** | - |  |  |  |  |  |
| (5) T1 Parental monitoring (mother) | -.23* | .15 | -.22* | .09 | - |  |  |  |  |
| (6) T1 Parental psychological control (adolescent) | -.28** | -.10 | .09 | -.33** | .13** | - |  |  |  |
| (7) T2 Internalizing symptoms (adolescent) | .23* | .03 | .24* | -.05 | -.20 | .08 | - |  |  |
| (8) T3 Internalizing symptoms (adolescent) | .44** | .05 | .11 | -.09 | -.19 | -.04 | .67** | - |  |
| (9) T4 Internalizing symptoms (adolescent) | .22** | -.08 | .31** | -.09 | -.14 | .07 | .59** | .74** | - |

*Note*: Observed scores for adolescent internalizing symptoms were computed at T2, T3, and T4. Adolescent gender was coded 0=boys, 1=girls. ** = *p* < .01; * = *p* < .05.

Table S11

*Correlations among study variables for United States- Latinx.*

|  | (1) | (2) | (3) | (4) | (5) | (6) | (7) | (8) | (9) |
| --- | --- | --- | --- | --- | --- | --- | --- | --- | --- |
| (1) Adolescent gender | - |  |  |  |  |  |  |  |  |
| (2) Family socioeconomic status (SES) | .13 | - |  |  |  |  |  |  |  |
| (3) T1 Adolescent negative emotionality (mother) | -.01 | -.24* | - |  |  |  |  |  |  |
| (4) T1 Adolescent effortful control (mother) | .04 | .05 | -.54** | - |  |  |  |  |  |
| (5) T1 Parental monitoring (mother) | -.12 | .00 | .16 | -.30* | - |  |  |  |  |
| (6) T1 Parental psychological control (adolescent) | .05 | .04 | -.30* | -.29* | -.12 | - |  |  |  |
| (7) T2 Internalizing symptoms (adolescent) | .13 | .32** | .32** | -.12 | -.15 | .09 | - |  |  |
| (8) T3 Internalizing symptoms (adolescent) | .32* | .13 | .47** | -.27 | .05 | .26 | .66** | - |  |
| (9) T4 Internalizing symptoms (adolescent) | .25 | .11 | .33* | -.22 | .08 | .13 | .52** | .67** | - |

*Note*: Observed scores for adolescent internalizing symptoms were computed at T2, T3, and T4. Adolescent gender was coded 0=boys, 1=girls. ** = *p* < .01; * = *p* < .05.

Table S12

*Covariates and within T1 relations across predictors from the conditional multiple group latent growth curve model for Colombia.*

|  | Estimated | S.E. | *p* value |
| --- | --- | --- | --- |
|  |  |  |  |
| Sex 🡪 Intercept | .19 | .02 | <.001 |
| Sex 🡪 Slope | .00 | -- | -- |
| Sex 🡪 Negative emotionality | .00 | -- | -- |
| Sex 🡪 Effortful control | .00 | -- | -- |
| Sex 🡪 Parental monitoring | .00 | -- | -- |
| Sex 🡪 Psychological control | .00 | -- | -- |
| Sex 🡪 Negative emotionality * psychological control | .00 | -- | -- |
| Sex 🡪 Effortful control * psychological control | .00 | -- | -- |
| Sex 🡪 Parental monitoring * psychological control | .00 | -- | -- |
| Ses 🡪 Intercept | .00 | -- | -- |
| Ses 🡪 Slope | .00 | -- | -- |
| Ses 🡪 Negative emotionality | **-.52** | **.07** | **<.001** |
| Ses 🡪 Effortful control | .14 | .04 | <.01 |
| Ses 🡪 Parental monitoring | -.12 | .04 | <.001 |
| Ses 🡪 Psychological control | -.03 | .03 | .29 |
| Ses 🡪 Negative emotionality * psychological control | .00 | -- | -- |
| Ses 🡪 Effortful control * psychological control | .00 | -- | -- |
| Ses 🡪 Parental monitoring * psychological control | .00 | -- | -- |
| Negative emotionality ↔ Effortful control | -.21 | .02 | <.001 |
| Negative emotionality ↔ Parental monitoring | **.04** | **.05** | **.44** |
| Negative emotionality ↔ Psychological control | .00 | -- | -- |
| Effortful control ↔ Parental monitoring | -.09 | .04 | .01 |
| Effortful control ↔ Psychological control | .00 | -- | -- |
| Parental monitoring ↔ Psychological control | .00 | -- | -- |
| Negative emotionality ↔ Negative emotionality * psychological control | **.06** | **.03** | **.02** |
| Negative emotionality ↔ Effortful control * psychological control | .00 | -- | -- |
| Negative emotionality ↔ Parental monitoring * psychological control | .00 | -- | -- |
| Effortful control ↔ Parental monitoring * psychological control | .00 | -- | -- |
| Effortful control ↔ Negative emotionality * psychological control | .00 | -- | -- |
| Effortful control ↔ Effortful control * psychological control | .00 | -- | -- |
| Parental monitoring ↔ Negative emotionality * psychological control | .00 | -- | -- |
| Parental monitoring ↔ Effortful control * psychological control | .00 | -- | -- |
| Parental monitoring ↔ Parental monitoring * psychological control | .00 | -- | -- |
| Psychological control ↔ Parental monitoring * psychological control | .00 | -- | -- |
| Psychological control ↔ Negative emotionality * psychological control | .00 | -- | -- |
| Psychological control ↔ Effortful control * psychological control | **.04** | **.02** | **.03** |
| Negative emotionality * psychological control ↔ Effortful control * psychological control | -.07 | .01 | <.001 |
| Negative emotionality * psychological control ↔ Parental monitoring * psychological control | .00 | -- | -- |
| Effortful control * psychological control ↔ Parental monitoring * psychological control | .00 | -- | -- |

*Note.* Unstandardized betas (->) with Standard Errors and correlation coefficients (↔) are reported. Bold indicate parameters varying across cultures. -- refers to those non-significant parameters that were constrained to be equal to zero to guarantee the model parsimony.

Table S13

*Covariates and within T1 relations across predictors from the conditional multiple group latent growth curve model for Italy-Naples.*

|  | Estimated | S.E. | *p* value |
| --- | --- | --- | --- |
|  |  |  |  |
| Sex 🡪 Intercept | .19 | .02 | <.001 |
| Sex 🡪 Slope | .00 | -- | -- |
| Sex 🡪 Negative emotionality | .00 | -- | -- |
| Sex 🡪 Effortful control | .00 | -- | -- |
| Sex 🡪 Parental monitoring | .00 | -- | -- |
| Sex 🡪 Psychological control | .00 | -- | -- |
| Sex 🡪 Negative emotionality * psychological control | .00 | -- | -- |
| Sex 🡪 Effortful control * psychological control | .00 | -- | -- |
| Sex 🡪 Parental monitoring * psychological control | .00 | -- | -- |
| Ses 🡪 Intercept | .00 | -- | -- |
| Ses 🡪 Slope | .00 | -- | -- |
| Ses 🡪 Negative emotionality | **-.37** | **.09** | **<.001** |
| Ses 🡪 Effortful control | .14 | .04 | <.01 |
| Ses 🡪 Parental monitoring | -.12 | .04 | <.001 |
| Ses 🡪 Psychological control | **-.28** | **.06** | **<.001** |
| Ses 🡪 Negative emotionality * psychological control | .00 | -- | -- |
| Ses 🡪 Effortful control * psychological control | .00 | -- | -- |
| Ses 🡪 Parental monitoring * psychological control | .00 | -- | -- |
| Negative emotionality ↔ Effortful control | -.21 | .02 | <.001 |
| Negative emotionality ↔ Parental monitoring | .00 | -- | -- |
| Negative emotionality ↔ Psychological control | .09 | .03 | <.01 |
| Effortful control ↔ Parental monitoring | .00 | -- | -- |
| Effortful control ↔ Psychological control | -.06 | .01 | <.001 |
| Parental monitoring ↔ Psychological control | .08 | .03 | .01 |
| Negative emotionality ↔ Negative emotionality * psychological control | **.03** | **.02** | **.23** |
| Negative emotionality ↔ Effortful control * psychological control | .00 | -- | -- |
| Negative emotionality ↔ Parental monitoring * psychological control | .00 | -- | -- |
| Effortful control ↔ Parental monitoring * psychological control | .00 | -- | -- |
| Effortful control ↔ Negative emotionality * psychological control | .00 | -- | -- |
| Effortful control ↔ Effortful control * psychological control | .00 | -- | -- |
| Parental monitoring ↔ Negative emotionality * psychological control | .00 | -- | -- |
| Parental monitoring ↔ Effortful control * psychological control | .00 | -- | -- |
| Parental monitoring ↔ Parental monitoring * psychological control | .00 | -- | -- |
| Psychological control ↔ Parental monitoring * psychological control | .00 | -- | -- |
| Psychological control ↔ Negative emotionality * psychological control | **.09** | **.02** | **<.001** |
| Psychological control ↔ Effortful control * psychological control | **-.03** | **.02** | **.08** |
| Negative emotionality * psychological control ↔ Effortful control * psychological control | **-.15** | **.03** | **<.001** |
| Negative emotionality * psychological control ↔ Parental monitoring * psychological control | .00 | -- | -- |
| Effortful control * psychological control ↔ Parental monitoring * psychological control | **.02** | **.01** | **.06** |

*Note.* Unstandardized betas (->) with Standard Errors and correlation coefficients (↔) are reported. Bold indicate parameters varying across cultures. -- refers to those non-significant parameters that were constrained to be equal to zero to guarantee the model parsimony.

Table S14

*Covariates and within T1 relations across predictors from the conditional multiple group latent growth curve model for Italy-Rome.*

|  | Estimated | S.E. | *p* value |
| --- | --- | --- | --- |
|  |  |  |  |
| Sex 🡪 Intercept | .19 | .02 | <.001 |
| Sex 🡪 Slope | .00 | -- | -- |
| Sex 🡪 Negative emotionality | .00 | -- | -- |
| Sex 🡪 Effortful control | .00 | -- | -- |
| Sex 🡪 Parental monitoring | .00 | -- | -- |
| Sex 🡪 Psychological control | .00 | -- | -- |
| Sex 🡪 Negative emotionality * psychological control | .00 | -- | -- |
| Sex 🡪 Effortful control * psychological control | .00 | -- | -- |
| Sex 🡪 Parental monitoring * psychological control | .00 | -- | -- |
| Ses 🡪 Intercept | .00 | -- | -- |
| Ses 🡪 Slope | .00 | -- | -- |
| Ses 🡪 Negative emotionality | -.07 | .05 | .16 |
| Ses 🡪 Effortful control | .14 | .04 | <.01 |
| Ses 🡪 Parental monitoring | -.12 | .04 | <.001 |
| Ses 🡪 Psychological control | -.03 | .03 | .29 |
| Ses 🡪 Negative emotionality * psychological control | .00 | -- | -- |
| Ses 🡪 Effortful control * psychological control | .00 | -- | -- |
| Ses 🡪 Parental monitoring * psychological control | .00 | -- | -- |
| Negative emotionality ↔ Effortful control | **-.07** | **.03** | **.01** |
| Negative emotionality ↔ Parental monitoring | .00 | -- | -- |
| Negative emotionality ↔ Psychological control | .00 | -- | -- |
| Effortful control ↔ Parental monitoring | .00 | -- | -- |
| Effortful control ↔ Psychological control | -.06 | .01 | <.001 |
| Parental monitoring ↔ Psychological control | **.02** | **.02** | **.46** |
| Negative emotionality ↔ Negative emotionality * psychological control | .03 | .02 | .34 |
| Negative emotionality ↔ Effortful control * psychological control | .01 | .01 | .52 |
| Negative emotionality ↔ Parental monitoring * psychological control | .00 | -- | -- |
| Effortful control ↔ Parental monitoring * psychological control | .00 | -- | -- |
| Effortful control ↔ Negative emotionality * psychological control | .02 | .01 | .09 |
| Effortful control ↔ Effortful control * psychological control | **-.02** | **.01** | **.19** |
| Parental monitoring ↔ Negative emotionality * psychological control | **-.02** | **.01** | **.05** |
| Parental monitoring ↔ Effortful control * psychological control | .00 | -- | -- |
| Parental monitoring ↔ Parental monitoring * psychological control | -.05 | -- | <.001 |
| Psychological control ↔ Parental monitoring * psychological control | **-.02** | **.01** | **.08** |
| Psychological control ↔ Negative emotionality * psychological control | .00 | .00 | -- |
| Psychological control ↔ Effortful control * psychological control | -.03 | .02 | .03 |
| Negative emotionality * psychological control ↔ Effortful control * psychological control | -.03 | .01 | <.001 |
| Negative emotionality * psychological control ↔ Parental monitoring * psychological control | .00 | -- | -- |
| Effortful control * psychological control ↔ Parental monitoring * psychological control | .00 | -- | -- |

*Note.* Unstandardized betas (->) with Standard Errors and correlation coefficients (↔) are reported. Bold indicate parameters varying across cultures. -- refers to those non-significant parameters that were constrained to be equal to zero to guarantee the model parsimony.

Table S15

*Covariates and within T1 relations across predictors from the conditional multiple group latent growth curve model for African American.*

|  | Estimated | S.E. | *p* value |
| --- | --- | --- | --- |
|  |  |  |  |
| Sex 🡪 Intercept | .19 | .02 | <.001 |
| Sex 🡪 Slope | .00 | -- | -- |
| Sex 🡪 Negative emotionality | .00 | -- | -- |
| Sex 🡪 Effortful control | .00 | -- | -- |
| Sex 🡪 Parental monitoring | .00 | -- | -- |
| Sex 🡪 Psychological control | .00 | -- | -- |
| Sex 🡪 Negative emotionality * psychological control | .00 | -- | -- |
| Sex 🡪 Effortful control * psychological control | .00 | -- | -- |
| Sex 🡪 Parental monitoring * psychological control | .00 | -- | -- |
| Ses 🡪 Intercept | .00 | -- | -- |
| Ses 🡪 Slope | .00 | -- | -- |
| Ses 🡪 Negative emotionality | -.07 | .05 | .16 |
| Ses 🡪 Effortful control | .14 | .04 | <.01 |
| Ses 🡪 Parental monitoring | -.12 | .04 | <.001 |
| Ses 🡪 Psychological control | -.03 | .03 | .29 |
| Ses 🡪 Negative emotionality * psychological control | .00 | -- | -- |
| Ses 🡪 Effortful control * psychological control | .00 | -- | -- |
| Ses 🡪 Parental monitoring * psychological control | .00 | -- | -- |
| Negative emotionality ↔ Effortful control | -.21 | .02 | <.001 |
| Negative emotionality ↔ Parental monitoring | .00 | -- | -- |
| Negative emotionality ↔ Psychological control | .00 | -- | -- |
| Effortful control ↔ Parental monitoring | .00 | -- | -- |
| Effortful control ↔ Psychological control | .00 | -- | -- |
| Parental monitoring ↔ Psychological control | .00 | -- | -- |
| Negative emotionality ↔ Negative emotionality * psychological control | **.04** | **.01** | **.01** |
| Negative emotionality ↔ Effortful control * psychological control | .00 | -- | -- |
| Negative emotionality ↔ Parental monitoring * psychological control | .00 | -- | -- |
| Effortful control ↔ Parental monitoring * psychological control | .00 | -- | -- |
| Effortful control ↔ Negative emotionality * psychological control | .00 | -- | -- |
| Effortful control ↔ Effortful control * psychological control | .00 | -- | -- |
| Parental monitoring ↔ Negative emotionality * psychological control | .00 | -- | -- |
| Parental monitoring ↔ Effortful control * psychological control | .00 | -- | -- |
| Parental monitoring ↔ Parental monitoring * psychological control | .00 | -- | -- |
| Psychological control ↔ Parental monitoring * psychological control | **.07** | **.01** | **<.001** |
| Psychological control ↔ Negative emotionality * psychological control | **-.07** | **.01** | **<.001** |
| Psychological control ↔ Effortful control * psychological control | .00 | -- | -- |
| Negative emotionality * psychological control ↔ Effortful control * psychological control | -.07 | .01 | <.001 |
| Negative emotionality * psychological control ↔ Parental monitoring * psychological control | .00 | -- | -- |
| Effortful control * psychological control ↔ Parental monitoring * psychological control | .00 | -- | -- |

*Note.* Unstandardized betas (->) with Standard Errors and correlation coefficients (↔) are reported. Bold indicate parameters varying across cultures. -- refers to those non-significant parameters that were constrained to be equal to zero to guarantee the model parsimony.

Table S16

*Covariates and within T1 relations across predictors from the conditional multiple group latent growth curve model for European American.*

|  | Estimated | S.E. | *p* value |
| --- | --- | --- | --- |
|  |  |  |  |
| Sex 🡪 Intercept | .19 | .02 | <.001 |
| Sex 🡪 Slope | .00 | -- | -- |
| Sex 🡪 Negative emotionality | .00 | -- | -- |
| Sex 🡪 Effortful control | .00 | -- | -- |
| Sex 🡪 Parental monitoring | .00 | -- | -- |
| Sex 🡪 Psychological control | .00 | -- | -- |
| Sex 🡪 Negative emotionality * psychological control | .00 | -- | -- |
| Sex 🡪 Effortful control * psychological control | .00 | -- | -- |
| Sex 🡪 Parental monitoring * psychological control | .00 | -- | -- |
| Ses 🡪 Intercept | .00 | -- | -- |
| Ses 🡪 Slope | .00 | -- | -- |
| Ses 🡪 Negative emotionality | -.07 | .05 | .16 |
| Ses 🡪 Effortful control | .14 | .04 | <.01 |
| Ses 🡪 Parental monitoring | **.15** | **.07** | **.02** |
| Ses 🡪 Psychological control | -.03 | .03 | .29 |
| Ses 🡪 Negative emotionality * psychological control | .00 | -- | -- |
| Ses 🡪 Effortful control * psychological control | .00 | -- | -- |
| Ses 🡪 Parental monitoring * psychological control | .00 | -- | -- |
| Negative emotionality ↔ Effortful control | -.21 | .02 | <.001 |
| Negative emotionality ↔ Parental monitoring | .10 | .03 | <.01 |
| Negative emotionality ↔ Psychological control | .00 | -- | -- |
| Effortful control ↔ Parental monitoring | .00 | -- | -- |
| Effortful control ↔ Psychological control | **-.06** | **.01** | **<.001** |
| Parental monitoring ↔ Psychological control | **.03** | **.02** | **.16** |
| Negative emotionality ↔ Negative emotionality * psychological control | **-.08** | **.03** | **.01** |
| Negative emotionality ↔ Effortful control * psychological control | .00 | -- | -- |
| Negative emotionality ↔ Parental monitoring * psychological control | **.06** | **.01** | **<.001** |
| Effortful control ↔ Parental monitoring * psychological control | **-.03** | **.01** | **<.01** |
| Effortful control ↔ Negative emotionality * psychological control | .00 | -- | -- |
| Effortful control ↔ Effortful control * psychological control | .00 | -- | -- |
| Parental monitoring ↔ Negative emotionality * psychological control | **.05** | **.02** | **<.01** |
| Parental monitoring ↔ Effortful control * psychological control | **-.02** | **.02** | **.25** |
| Parental monitoring ↔ Parental monitoring * psychological control | -.05 | .01 | <.001 |
| Psychological control ↔ Parental monitoring * psychological control | **.02** | **.01** | **.04** |
| Psychological control ↔ Negative emotionality * psychological control | .00 | -- | -- |
| Psychological control ↔ Effortful control * psychological control | .00 | -- | -- |
| Negative emotionality * psychological control ↔ Effortful control * psychological control | -.07 | .01 | <.001 |
| Negative emotionality * psychological control ↔ Parental monitoring * psychological control | **-.03** | **.01** | **<.01** |
| Effortful control * psychological control ↔ Parental monitoring * psychological control | **-.02** | **.01** | **.04** |

*Note.* Unstandardized betas (->) with Standard Errors and correlation coefficients (↔) are reported. Bold indicate parameters varying across cultures. -- refers to those non-significant parameters that were constrained to be equal to zero to guarantee the model parsimony.

Table S17

*Covariates and within T1 relations across predictors from the conditional multiple group latent growth curve model for Latinx.*

|  | Estimated | S.E. | *p* value |
| --- | --- | --- | --- |
|  |  |  |  |
| Sex 🡪 Intercept | .19 | .02 | <.001 |
| Sex 🡪 Slope | .00 | -- | -- |
| Sex 🡪 Negative emotionality | .00 | -- | -- |
| Sex 🡪 Effortful control | .00 | -- | -- |
| Sex 🡪 Parental monitoring | .00 | -- | -- |
| Sex 🡪 Psychological control | .00 | -- | -- |
| Sex 🡪 Negative emotionality * psychological control | .00 | -- | -- |
| Sex 🡪 Effortful control * psychological control | .00 | -- | -- |
| Sex 🡪 Parental monitoring * psychological control | .00 | -- | -- |
| Ses 🡪 Intercept | .00 | -- | -- |
| Ses 🡪 Slope | .00 | -- | -- |
| Ses 🡪 Negative emotionality | -.07 | .05 | .16 |
| Ses 🡪 Effortful control | .14 | .04 | <.01 |
| Ses 🡪 Parental monitoring | -.12 | .04 | <.001 |
| Ses 🡪 Psychological control | -.03 | .03 | .29 |
| Ses 🡪 Negative emotionality * psychological control | .00 | -- | -- |
| Ses 🡪 Effortful control * psychological control | .00 | -- | -- |
| Ses 🡪 Parental monitoring * psychological control | .00 | -- | -- |
| Negative emotionality ↔ Effortful control | -.21 | .02 | <.001 |
| Negative emotionality ↔ Parental monitoring | .00 | -- | -- |
| Negative emotionality ↔ Psychological control | .00 | -- | -- |
| Effortful control ↔ Parental monitoring | **-.09** | **.04** | **.01** |
| Effortful control ↔ Psychological control | -.06 | .01 | <.001 |
| Parental monitoring ↔ Psychological control | .00 | -- | -- |
| Negative emotionality ↔ Negative emotionality * psychological control | -.03 | .01 | .03 |
| Negative emotionality ↔ Effortful control * psychological control | .01 | .01 | .52 |
| Negative emotionality ↔ Parental monitoring * psychological control | .00 | -- | -- |
| Effortful control ↔ Parental monitoring * psychological control | .00 | -- | -- |
| Effortful control ↔ Negative emotionality * psychological control | .02 | .01 | .08 |
| Effortful control ↔ Effortful control * psychological control | **.05** | **.02** | **<.01** |
| Parental monitoring ↔ Negative emotionality * psychological control | .00 | -- | -- |
| Parental monitoring ↔ Effortful control * psychological control | **-.04** | **.02** | **.06** |
| Parental monitoring ↔ Parental monitoring * psychological control | .19 | .05 | <.001 |
| Psychological control ↔ Parental monitoring * psychological control | **-.00** | **.01** | **.87** |
| Psychological control ↔ Negative emotionality * psychological control | .00 | -- | -- |
| Psychological control ↔ Effortful control * psychological control | .00 | -- | -- |
| Negative emotionality * psychological control ↔ Effortful control * psychological control | **-.03** | **.01** | **<.01** |
| Negative emotionality * psychological control ↔ Parental monitoring * psychological control | .00 | -- | -- |
| Effortful control * psychological control ↔ Parental monitoring * psychological control | **-.01** | **.01** | **.19** |

*Note.* Unstandardized betas (->) with Standard Errors and correlation coefficients (↔) are reported. Bold indicate parameters varying across cultures. -- refers to those non-significant parameters that were constrained to be equal to zero to guarantee the model parsimony.
