# Supplementary material for: Development of internalizing symptoms during adolescence in three countries: the role of temperament and parenting behaviors
Source: Eur Child Adolesc Psychiatry. Author manuscript; Available in PMC 2023 Jun 1. (PMC8610087; doi:10.1007/s00787-021-01725-6)
Supplement: Supplementary file 1 [file NIHMS1754949-supplement-Supplemental_Material.pdf]

- Supplemental Material

Table S1

Sample sizes differentiated by site, time, and reporter.

		Time 1	Time 2	Time 3	Time 4
**Colombia**		Adolescents *n*=88<br>Mothers *n*=87	*n*=83	*n*=76	*n*=77
**Italy**	**Naples**	Adolescents *n*=90<br>Mothers *n*=90	*n*=88	*n*=83	*n*=85
	**Rome**	Adolescents *n*=100<br>Mothers *n*=102	*n*=96	*n*=100	*n*=99
**United States**	**African American**	Adolescents *n*=92<br>Mothers *n*=87	*n*=88	*n*=86	*n*=85
	**European American**	Adolescents *n*=97<br>Mothers *n*=94	*n*=92	*n*=88	*n*=88
	**Latinx**	Adolescents *n*=77<br>Mothers *n*=70	*n*=71	*n*=57	*n*=60

Note: Only adolescents reported at Time 2 through Time 4.

Table S2

Time 1 Sociodemographic Variables

	Colombia		Italy				United States						
			Rome		Naples		European American		African American		Latinx		
	M	SD	M	SD	M	SD	M	SD	M	SD	M	SD	
**Maternal years of education-Time 1**	10.26[a]	5.46	14.10[b]	4.04	10.34[a]	4.45	16.88[c]	2.75	13.58[b]	2.36	10.41[a]	4.16	F(5, 536)=43.22**
**Marital status (%) – Time 1**													F(5, 521)=17.87**
**Married**	69.3		75.5		83		81.4		37.2		55.7		
**Remarried**	0		0		2.2		2.1		1.2		2.9		
**Divorced**	0		1		1.1		7.2		9.3		7.1		
**Separated**	3.4		11.1		6.8		2.1		7		7.1		
**Widowed**	0		2		2.3		2.1		1.2		0		
**Cohabiting**	22.7		3.1		2.3		2.1		7		18.6		
**Never married**	4.5		7.1		2.3		3.1		37.2		8.6		
**Number of siblings**	2.10[a]	1.00	1.95[a]	.80	2.00[a]	.71	2.29[ab]	.97	2.25[ab]	1.27	2.57[b]	1.31	F(5, 527)=3.96**

Note. *=*p*<0.05; **=*p*<0.01. Superscripts indicate results of the Tukey Post-Hoc test. The groups with the same letter are statistically equal and different from the groups with different letters.

Table S3

Descriptive statistics for study variables within the total sample.

Time Variable (Reporter)	Mean	SD	Skewness	Kurtosis
T1 Adolescent negative emotionality (mother)	2.40	.74	.45	-.27
T1 Adolescent effortful control (mother)	3.48	.64	-.31	-.16
T1 Parental monitoring (mother)	-.00	.63	-1.38	2.27
T1 Parental psychological control (adolescent)	2.43	.51	.26	-.00
T2 Internalizing symptoms (adolescent)	.44	.30	.86	.75
T3 Internalizing symptoms (adolescent)	.51	.36	.97	.80
T4 Internalizing symptoms (adolescent)	.47	.31	.72	.19

Table S4

Correlations among study variables for the total sample (observed scores).

Variable (reporter)	(1)	(2)	(3)	(4)	(5)	(6)	(7)	(8)	(9)
(1) Adolescent gender	-								
(2) Family socioeconomic status (SES)	-.04	-							
(3) T1 Adolescent negative emotionality (mother)	.04	-.27**	-						
(4) T1 Adolescent effortful control (mother)	.09*	.14**	-.47**	-					
(5) T1 Parental monitoring (mother)	-.07	-.06	.05	.14**	-				
(6) T1 Parental psychological control (adolescent)	-.09*	-.18**	.14**	-.19**	.07	-			
(7) T2 Internalizing symptoms (adolescent)	.27**	.04	.21**	-.14**	-.10*	-.07	-		
(8) T3 Internalizing symptoms (adolescent)	.40**	.03	.19**	-.08	-.14**	.03	.62**	-	
(9) T4 Internalizing symptoms (adolescent)	.32**	-.03	.24**	-.10*	-.17**	.07	.57**	.74**	-

Note: Observed scores for adolescent internalizing symptoms were computed at T2, T3, and T4. Adolescent gender was coded 0=boys, 1=girls. ** = $p < .01$; * = $p < .05$.

Table S5

Descriptive statistics for study variables reported separately by cultural group.

	Colombia		Italy				United States					
			Naples		Rome		African American		European American		Latinx	
**Time Variable (reporter)**	**Mean**	**SD**	**Mean**	**SD**	**Mean**	**SD**	**Mean**	**SD**	**Mean**	**SD**	**Mean**	**SD**
T1 Adolescent negative emotionality (mother)	2.51	.87	2.60	.77	2.38	.59	2.21	.59	2.35	.71	2.31	.81
T1 Adolescent effortful control (mother)	3.54	.73	3.39	.63	3.45	.54	3.40	.54	3.56	.67	3.58	.61
T1 Parental monitoring (mother)	.01	.78	-.14	.61	-.17	.55	.38	.55	-.00	.43	-.06	.87
T1 Parental psychological control (adolescent)	2.41	.53	2.48	.52	2.37	.50	2.49	.50	2.27	.51	2.60	.41
T2 Internalizing symptoms (adolescent)	.49	.30	.46	.29	.46	.27	.41	.27	.46	.33	.32	.23
T3 Internalizing symptoms (adolescent)	.61	.41	.54	.34	.53	.31	.53	.31	.56	.39	.40	.29
T4 Internalizing symptoms (adolescent)	.55	.30	.50	.27	.50	.30	.50	.30	.49	.35	.40	.27

Table S6

Correlations among study variables for Colombia.

Variable (reporter)	(1)	(2)	(3)	(4)	(5)	(6)	(7)	(8)	(9)
(1) Adolescent gender	-								
(2) Family socioeconomic status (SES)	.03	-							
(3) T1 Adolescent negative emotionality (mother)	.08	-.61**	-						
(4) T1 Adolescent effortful control (mother)	-.09	.29**	-.58**	-					
(5) T1 Parental monitoring (mother)	-.08	-.33**	.27*	-.24*	-				
(6) T1 Parental psychological control (adolescent)	-.14	.02	.05	-.07	.06	-			
(7) T2 Internalizing symptoms (adolescent)	.39**	-.07	.17	-.15	-.08	.11	-		
(8) T3 Internalizing symptoms (adolescent)	.56**	-.11	.15	-.18	-.06	.14	.65**	-	
(9) T4 Internalizing symptoms (adolescent)	.42**	-.08	.26*	-.16	-.06	.19	.62**	.74**	-

Note: Observed scores for adolescent internalizing symptoms were computed at T2, T3, and T4. Adolescent gender was coded 0=boys, 1=girls. ** = $p < .01$; * = $p < .05$.

Table S7

Correlations among study variables for Italy-Naples.

	(1)	(2)	(3)	(4)	(5)	(6)	(7)	(8)	(9)
(1) Adolescent gender	-								
(2) Family socioeconomic status (SES)	.09	-							
(3) T1 Adolescent negative emotionality (mother)	-.14	-.39**	-						
(4) T1 Adolescent effortful control (mother)	.21*	.17	-.45**	-					
(5) T1 Parental monitoring (mother)	.08	.05	.01	.04	-				
(6) T1 Parental psychological control (adolescent)	-.09	-.45**	-.42**	-.25*	.27*	-			
(7) T2 Internalizing symptoms (adolescent)	.33**	.01	.06	-.21	-.21	.00	-		
(8) T3 Internalizing symptoms (adolescent)	.39**	.07	.05	-.03	-.25*	.06	.66**	-	
(9) T4 Internalizing symptoms (adolescent)	.31**	-.05	.11	-.00	-.32**	.02	.68**	.84**	-

Note: Observed scores for adolescent internalizing symptoms were computed at T2, T3, and T4. Adolescent gender was coded 0=boys, 1=girls. ** = $p < .01$; * = $p < .05$.

Table S8

Correlations among study variables for Italy- Rome.

	(1)	(2)	(3)	(4)	(5)	(6)	(7)	(8)	(9)
(1) Adolescent gender	-								
(2) Family socioeconomic status (SES)	-.08	-							
(3) T1 Adolescent negative emotionality (mother)	.10	-.06	-						
(4) T1 Adolescent effortful control (mother)	.06	.14	-.31**	-					
(5) T1 Parental monitoring (mother)	-.03	-.16	.04	-.05	-				
(6) T1 Parental psychological control (adolescent)	-.09*	-.16	.08	-.21**	.10	-			
(7) T2 Internalizing symptoms (adolescent)	.36**	.06	.28**	-.19	-.23*	.15	-		
(8) T3 Internalizing symptoms (adolescent)	.44**	.00	.26**	-.21*	-.18	.13	.63**	-	
(9) T4 Internalizing symptoms (adolescent)	.46**	-.02	.18	-.20*	-.12*	.17	.59**	.75**	-

Note: Observed scores for adolescent internalizing symptoms were computed at T2, T3, and T4. Adolescent gender was coded 0=boys, 1=girls. ** = $p < .01$; * = $p < .05$.

Table S9

Correlations among study variables for United States-African American.

	(1)	(2)	(3)	(4)	(5)	(6)	(7)	(8)	(9)
(1) Adolescent gender	-								
(2) Family socioeconomic status (SES)	.01	-							
(3) T1 Adolescent negative emotionality (mother)	.16	-.15	-						
(4) T1 Adolescent effortful control (mother)	.10	.23**	-.49**	-					
(5) T1 Parental monitoring (mother)	-.11	-.27**	.03	-.01	-				
(6) T1 Parental psychological control (adolescent)	-.12	-.00	.03	-.09	.10	-			
(7) T2 Internalizing symptoms (adolescent)	.21	-.08	.22**	-.12	-.01	.19	-		
(8) T3 Internalizing symptoms (adolescent)	.29**	.08	.12	-.03	-.06	.04	.45**	-	
(9) T4 Internalizing symptoms (adolescent)	.32**	.01	.17	.02	-.15	.04	.41**	.68**	-

Note: Observed scores for adolescent internalizing symptoms were computed at T2, T3, and T4. Adolescent gender was coded 0=boys, 1=girls. ** = $p < .01$; * = $p < .05$.

Table S10

Correlations among study variables for United States-European American.

	(1)	(2)	(3)	(4)	(5)	(6)	(7)	(8)	(9)
(1) Adolescent gender	-								
(2) Family socioeconomic status (SES)	-.02	-							
(3) T1 Adolescent negative emotionality (mother)	.04	.07	-						
(4) T1 Adolescent effortful control (mother)	.25*	.10	-.43**	-					
(5) T1 Parental monitoring (mother)	-.23*	.15	-.22*	.09	-				
(6) T1 Parental psychological control (adolescent)	-.28**	-.10	.09	-.33**	.13**	-			
(7) T2 Internalizing symptoms (adolescent)	.23*	.03	.24*	-.05	-.20	.08	-		
(8) T3 Internalizing symptoms (adolescent)	.44**	.05	.11	-.09	-.19	-.04	.67**	-	
(9) T4 Internalizing symptoms (adolescent)	.22**	-.08	.31**	-.09	-.14	.07	.59**	.74**	-

Note: Observed scores for adolescent internalizing symptoms were computed at T2, T3, and T4. Adolescent gender was coded 0=boys, 1=girls. ** = $p < .01$; * = $p < .05$.

Table S11

Correlations among study variables for United States- Latinx.

	(1)	(2)	(3)	(4)	(5)	(6)	(7)	(8)	(9)
(1) Adolescent gender	-								
(2) Family socioeconomic status (SES)	.13	-							
(3) T1 Adolescent negative emotionality (mother)	-.01	-.24*	-						
(4) T1 Adolescent effortful control (mother)	.04	.05	-.54**	-					
(5) T1 Parental monitoring (mother)	-.12	.00	.16	-.30*	-				
(6) T1 Parental psychological control (adolescent)	.05	.04	-.30*	-.29*	-.12	-			
(7) T2 Internalizing symptoms (adolescent)	.13	.32**	.32**	-.12	-.15	.09	-		
(8) T3 Internalizing symptoms (adolescent)	.32*	.13	.47**	-.27	.05	.26	.66**	-	
(9) T4 Internalizing symptoms (adolescent)	.25	.11	.33*	-.22	.08	.13	.52**	.67**	-

Note: Observed scores for adolescent internalizing symptoms were computed at T2, T3, and T4. Adolescent gender was coded 0=boys, 1=girls. ** = p < .01; * = p < .05.

Table S12

Covariates and within T1 relations across predictors from the conditional multiple group latent growth curve model for Colombia.

	Estimated	S.E.	*p* value
Sex → Intercept	.19	.02	<.001
Sex → Slope	.00	--	--
Sex → Negative emotionality	.00	--	--
Sex → Effortful control	.00	--	--
Sex → Parental monitoring	.00	--	--
Sex → Psychological control	.00	--	--
Sex → Negative emotionality * psychological control	.00	--	--
Sex → Effortful control * psychological control	.00	--	--
Sex → Parental monitoring * psychological control	.00	--	--
Ses → Intercept	.00	--	--
Ses → Slope	.00	--	--
Ses → Negative emotionality	**-.52**	**.07**	**<.001**
Ses → Effortful control	.14	.04	<.01
Ses → Parental monitoring	-.12	.04	<.001
Ses → Psychological control	-.03	.03	.29
Ses → Negative emotionality * psychological control	.00	--	--
Ses → Effortful control * psychological control	.00	--	--
Ses → Parental monitoring * psychological control	.00	--	--
Negative emotionality ↔ Effortful control	-.21	.02	<.001
Negative emotionality ↔ Parental monitoring	**.04**	**.05**	**.44**
Negative emotionality ↔ Psychological control	.00	--	--
Effortful control ↔ Parental monitoring	-.09	.04	.01
Effortful control ↔ Psychological control	.00	--	--
Parental monitoring ↔ Psychological control	.00	--	--
Negative emotionality ↔ Negative emotionality * psychological control	**.06**	**.03**	**.02**
Negative emotionality ↔ Effortful control * psychological control	.00	--	--
Negative emotionality ↔ Parental monitoring * psychological control	.00	--	--
Effortful control ↔ Parental monitoring * psychological control	.00	--	--
Effortful control ↔ Negative emotionality * psychological control	.00	--	--
Effortful control ↔ Effortful control * psychological control	.00	--	--
Parental monitoring ↔ Negative emotionality * psychological control	.00	--	--
Parental monitoring ↔ Effortful control * psychological control	.00	--	--

Parental monitoring ↔ Parental monitoring * psychological control	.00	--	--
Psychological control ↔ Parental monitoring * psychological control	.00	--	--
Psychological control ↔ Negative emotionality * psychological control	.00	--	--
Psychological control ↔ Effortful control * psychological control	**.04**	**.02**	**.03**
Negative emotionality * psychological control ↔ Effortful control * psychological control	-.07	.01	<.001
Negative emotionality * psychological control ↔ Parental monitoring * psychological control	.00	--	--
Effortful control * psychological control ↔ Parental monitoring * psychological control	.00	--	--

Note. Unstandardized betas (->) with Standard Errors and correlation coefficients (↔) are reported. Bold indicate parameters varying across cultures. -- refers to those non-significant parameters that were constrained to be equal to zero to guarantee the model parsimony.

Table S13

Covariates and within T1 relations across predictors from the conditional multiple group latent growth curve model for Italy-Naples.

	Estimated	S.E.	*p* value
Sex → Intercept	.19	.02	<.001
Sex → Slope	.00	--	--
Sex → Negative emotionality	.00	--	--
Sex → Effortful control	.00	--	--
Sex → Parental monitoring	.00	--	--
Sex → Psychological control	.00	--	--
Sex → Negative emotionality * psychological control	.00	--	--
Sex → Effortful control * psychological control	.00	--	--
Sex → Parental monitoring * psychological control	.00	--	--
Ses → Intercept	.00	--	--
Ses → Slope	.00	--	--
Ses → Negative emotionality	**-.37**	**.09**	**<.001**
Ses → Effortful control	.14	.04	<.01
Ses → Parental monitoring	-.12	.04	<.001
Ses → Psychological control	**-.28**	**.06**	**<.001**
Ses → Negative emotionality * psychological control	.00	--	--
Ses → Effortful control * psychological control	.00	--	--
Ses → Parental monitoring * psychological control	.00	--	--
Negative emotionality ↔ Effortful control	-.21	.02	<.001
Negative emotionality ↔ Parental monitoring	.00	--	--
Negative emotionality ↔ Psychological control	.09	.03	<.01
Effortful control ↔ Parental monitoring	.00	--	--
Effortful control ↔ Psychological control	-.06	.01	<.001
Parental monitoring ↔ Psychological control	.08	.03	.01
Negative emotionality ↔ Negative emotionality * psychological control	**.03**	**.02**	**.23**
Negative emotionality ↔ Effortful control * psychological control	.00	--	--
Negative emotionality ↔ Parental monitoring * psychological control	.00	--	--
Effortful control ↔ Parental monitoring * psychological control	.00	--	--
Effortful control ↔ Negative emotionality * psychological control	.00	--	--
Effortful control ↔ Effortful control * psychological control	.00	--	--
Parental monitoring ↔ Negative emotionality * psychological control	.00	--	--
Parental monitoring ↔ Effortful control * psychological control	.00	--	--

Parental monitoring ↔ Parental monitoring * psychological control	.00	--	--
Psychological control ↔ Parental monitoring * psychological control	.00	--	--
Psychological control ↔ Negative emotionality * psychological control	**.09**	**.02**	**<.001**
Psychological control ↔ Effortful control * psychological control	**-.03**	**.02**	**.08**
Negative emotionality * psychological control ↔ Effortful control * psychological control	**-.15**	**.03**	**<.001**
Negative emotionality * psychological control ↔ Parental monitoring * psychological control	.00	--	--
Effortful control * psychological control ↔ Parental monitoring * psychological control	**.02**	**.01**	**.06**

Note. Unstandardized betas (->) with Standard Errors and correlation coefficients (↔) are reported. Bold indicate parameters varying across cultures. -- refers to those non-significant parameters that were constrained to be equal to zero to guarantee the model parsimony.

Table S14

Covariates and within T1 relations across predictors from the conditional multiple group latent growth curve model for Italy-Rome.

	Estimated	S.E.	*p* value
Sex → Intercept	.19	.02	<.001
Sex → Slope	.00	--	--
Sex → Negative emotionality	.00	--	--
Sex → Effortful control	.00	--	--
Sex → Parental monitoring	.00	--	--
Sex → Psychological control	.00	--	--
Sex → Negative emotionality * psychological control	.00	--	--
Sex → Effortful control * psychological control	.00	--	--
Sex → Parental monitoring * psychological control	.00	--	--
Ses → Intercept	.00	--	--
Ses → Slope	.00	--	--
Ses → Negative emotionality	-.07	.05	.16
Ses → Effortful control	.14	.04	<.01
Ses → Parental monitoring	-.12	.04	<.001
Ses → Psychological control	-.03	.03	.29
Ses → Negative emotionality * psychological control	.00	--	--
Ses → Effortful control * psychological control	.00	--	--
Ses → Parental monitoring * psychological control	.00	--	--
Negative emotionality ↔ Effortful control	**-.07**	**.03**	**.01**
Negative emotionality ↔ Parental monitoring	.00	--	--
Negative emotionality ↔ Psychological control	.00	--	--
Effortful control ↔ Parental monitoring	.00	--	--
Effortful control ↔ Psychological control	-.06	.01	<.001
Parental monitoring ↔ Psychological control	**.02**	**.02**	**.46**
Negative emotionality ↔ Negative emotionality * psychological control	.03	.02	.34
Negative emotionality ↔ Effortful control * psychological control	.01	.01	.52
Negative emotionality ↔ Parental monitoring * psychological control	.00	--	--
Effortful control ↔ Parental monitoring * psychological control	.00	--	--
Effortful control ↔ Negative emotionality * psychological control	.02	.01	.09
Effortful control ↔ Effortful control * psychological control	**-.02**	**.01**	**.19**
Parental monitoring ↔ Negative emotionality * psychological control	**-.02**	**.01**	**.05**
Parental monitoring ↔ Effortful control * psychological control	.00	--	--

Parental monitoring ↔ Parental monitoring * psychological control	-.05	--	<.001
Psychological control ↔ Parental monitoring * psychological control	**-.02**	**.01**	**.08**
Psychological control ↔ Negative emotionality * psychological control	.00	.00	--
Psychological control ↔ Effortful control * psychological control	-.03	.02	.03
Negative emotionality * psychological control ↔ Effortful control * psychological control	-.03	.01	<.001
Negative emotionality * psychological control ↔ Parental monitoring * psychological control	.00	--	--
Effortful control * psychological control ↔ Parental monitoring * psychological control	.00	--	--

Note. Unstandardized betas (->) with Standard Errors and correlation coefficients (↔) are reported. Bold indicate parameters varying across cultures. -- refers to those non-significant parameters that were constrained to be equal to zero to guarantee the model parsimony.

Table S15

Covariates and within T1 relations across predictors from the conditional multiple group latent growth curve model for African American.

	Estimated	S.E.	*p* value
Sex → Intercept	.19	.02	<.001
Sex → Slope	.00	--	--
Sex → Negative emotionality	.00	--	--
Sex → Effortful control	.00	--	--
Sex → Parental monitoring	.00	--	--
Sex → Psychological control	.00	--	--
Sex → Negative emotionality * psychological control	.00	--	--
Sex → Effortful control * psychological control	.00	--	--
Sex → Parental monitoring * psychological control	.00	--	--
Ses → Intercept	.00	--	--
Ses → Slope	.00	--	--
Ses → Negative emotionality	-.07	.05	.16
Ses → Effortful control	.14	.04	<.01
Ses → Parental monitoring	-.12	.04	<.001
Ses → Psychological control	-.03	.03	.29
Ses → Negative emotionality * psychological control	.00	--	--
Ses → Effortful control * psychological control	.00	--	--
Ses → Parental monitoring * psychological control	.00	--	--
Negative emotionality ↔ Effortful control	-.21	.02	<.001
Negative emotionality ↔ Parental monitoring	.00	--	--
Negative emotionality ↔ Psychological control	.00	--	--
Effortful control ↔ Parental monitoring	.00	--	--
Effortful control ↔ Psychological control	.00	--	--
Parental monitoring ↔ Psychological control	.00	--	--
Negative emotionality ↔ Negative emotionality * psychological control	**.04**	**.01**	**.01**
Negative emotionality ↔ Effortful control * psychological control	.00	--	--
Negative emotionality ↔ Parental monitoring * psychological control	.00	--	--
Effortful control ↔ Parental monitoring * psychological control	.00	--	--
Effortful control ↔ Negative emotionality * psychological control	.00	--	--
Effortful control ↔ Effortful control * psychological control	.00	--	--
Parental monitoring ↔ Negative emotionality * psychological control	.00	--	--
Parental monitoring ↔ Effortful control * psychological control	.00	--	--

Parental monitoring ↔ Parental monitoring * psychological control	.00	--	--
Psychological control ↔ Parental monitoring * psychological control	**.07**	**.01**	**<.001**
Psychological control ↔ Negative emotionality * psychological control	**-.07**	**.01**	**<.001**
Psychological control ↔ Effortful control * psychological control	.00	--	--
Negative emotionality * psychological control ↔ Effortful control * psychological control	-.07	.01	<.001
Negative emotionality * psychological control ↔ Parental monitoring * psychological control	.00	--	--
Effortful control * psychological control ↔ Parental monitoring * psychological control	.00	--	--

Note. Unstandardized betas (->) with Standard Errors and correlation coefficients (↔) are reported. Bold indicate parameters varying across cultures. -- refers to those non-significant parameters that were constrained to be equal to zero to guarantee the model parsimony.

Table S16

Covariates and within T1 relations across predictors from the conditional multiple group latent growth curve model for European American.

	Estimated	S.E.	*p* value
Sex → Intercept	.19	.02	<.001
Sex → Slope	.00	--	--
Sex → Negative emotionality	.00	--	--
Sex → Effortful control	.00	--	--
Sex → Parental monitoring	.00	--	--
Sex → Psychological control	.00	--	--
Sex → Negative emotionality * psychological control	.00	--	--
Sex → Effortful control * psychological control	.00	--	--
Sex → Parental monitoring * psychological control	.00	--	--
Ses → Intercept	.00	--	--
Ses → Slope	.00	--	--
Ses → Negative emotionality	-.07	.05	.16
Ses → Effortful control	.14	.04	<.01
Ses → Parental monitoring	**.15**	**.07**	**.02**
Ses → Psychological control	-.03	.03	.29
Ses → Negative emotionality * psychological control	.00	--	--
Ses → Effortful control * psychological control	.00	--	--
Ses → Parental monitoring * psychological control	.00	--	--
Negative emotionality ↔ Effortful control	-.21	.02	<.001
Negative emotionality ↔ Parental monitoring	.10	.03	<.01
Negative emotionality ↔ Psychological control	.00	--	--
Effortful control ↔ Parental monitoring	.00	--	--
Effortful control ↔ Psychological control	**-.06**	**.01**	**<.001**
Parental monitoring ↔ Psychological control	**.03**	**.02**	**.16**
Negative emotionality ↔ Negative emotionality * psychological control	**-.08**	**.03**	**.01**
Negative emotionality ↔ Effortful control * psychological control	.00	--	--
Negative emotionality ↔ Parental monitoring * psychological control	**.06**	**.01**	**<.001**
Effortful control ↔ Parental monitoring * psychological control	**-.03**	**.01**	**<.01**
Effortful control ↔ Negative emotionality * psychological control	.00	--	--
Effortful control ↔ Effortful control * psychological control	.00	--	--
Parental monitoring ↔ Negative emotionality * psychological control	**.05**	**.02**	**<.01**
Parental monitoring ↔ Effortful control * psychological control	**-.02**	**.02**	**.25**

Parental monitoring ↔ Parental monitoring * psychological control	-.05	.01	<.001
Psychological control ↔ Parental monitoring * psychological control	**.02**	**.01**	**.04**
Psychological control ↔ Negative emotionality * psychological control	.00	--	--
Psychological control ↔ Effortful control * psychological control	.00	--	--
Negative emotionality * psychological control ↔ Effortful control * psychological control	-.07	.01	<.001
Negative emotionality * psychological control ↔ Parental monitoring * psychological control	**-.03**	**.01**	**<.01**
Effortful control * psychological control ↔ Parental monitoring * psychological control	**-.02**	**.01**	**.04**

Note. Unstandardized betas (->) with Standard Errors and correlation coefficients (↔) are reported. Bold indicate parameters varying across cultures. -- refers to those non-significant parameters that were constrained to be equal to zero to guarantee the model parsimony.

Table S17

Covariates and within T1 relations across predictors from the conditional multiple group latent growth curve model for Latinx.

	Estimated	S.E.	*p* value
Sex → Intercept	.19	.02	<.001
Sex → Slope	.00	--	--
Sex → Negative emotionality	.00	--	--
Sex → Effortful control	.00	--	--
Sex → Parental monitoring	.00	--	--
Sex → Psychological control	.00	--	--
Sex → Negative emotionality * psychological control	.00	--	--
Sex → Effortful control * psychological control	.00	--	--
Sex → Parental monitoring * psychological control	.00	--	--
Ses → Intercept	.00	--	--
Ses → Slope	.00	--	--
Ses → Negative emotionality	-.07	.05	.16
Ses → Effortful control	.14	.04	<.01
Ses → Parental monitoring	-.12	.04	<.001
Ses → Psychological control	-.03	.03	.29
Ses → Negative emotionality * psychological control	.00	--	--
Ses → Effortful control * psychological control	.00	--	--
Ses → Parental monitoring * psychological control	.00	--	--
Negative emotionality ↔ Effortful control	-.21	.02	<.001
Negative emotionality ↔ Parental monitoring	.00	--	--
Negative emotionality ↔ Psychological control	.00	--	--
Effortful control ↔ Parental monitoring	**-.09**	**.04**	**.01**
Effortful control ↔ Psychological control	-.06	.01	<.001
Parental monitoring ↔ Psychological control	.00	--	--
Negative emotionality ↔ Negative emotionality * psychological control	-.03	.01	.03
Negative emotionality ↔ Effortful control * psychological control	.01	.01	.52
Negative emotionality ↔ Parental monitoring * psychological control	.00	--	--
Effortful control ↔ Parental monitoring * psychological control	.00	--	--
Effortful control ↔ Negative emotionality * psychological control	.02	.01	.08
Effortful control ↔ Effortful control * psychological control	**.05**	**.02**	**<.01**
Parental monitoring ↔ Negative emotionality * psychological control	.00	--	--
Parental monitoring ↔ Effortful control * psychological control	**-.04**	**.02**	**.06**

Parental monitoring ↔ Parental monitoring * psychological control	.19	.05	<.001
Psychological control ↔ Parental monitoring * psychological control	**-.00**	**.01**	**.87**
Psychological control ↔ Negative emotionality * psychological control	.00	--	--
Psychological control ↔ Effortful control * psychological control	.00	--	--
Negative emotionality * psychological control ↔ Effortful control * psychological control	**-.03**	**.01**	**<.01**
Negative emotionality * psychological control ↔ Parental monitoring * psychological control	.00	--	--
Effortful control * psychological control ↔ Parental monitoring * psychological control	**-.01**	**.01**	**.19**

Note. Unstandardized betas (->) with Standard Errors and correlation coefficients (↔) are reported. Bold indicate parameters varying across cultures. -- refers to those non-significant parameters that were constrained to be equal to zero to guarantee the model parsimony.
